# Dual-expression system for blue fluorescent protein optimization

**DOI:** 10.1038/s41598-022-13214-0

**Published:** 2022-06-17

**Authors:** Stavrini Papadaki, Xinyue Wang, Yangdong Wang, Hanbin Zhang, Su Jia, Shuhong Liu, Minghan Yang, Dongdong Zhang, Jie-Min Jia, Reinhard W. Köster, Kazuhiko Namikawa, Kiryl D. Piatkevich

**Affiliations:** 1 School of Life Sciences, Westlake University, Hangzhou, Zhejiang Province China; 2 Westlake Laboratory of Life Sciences and Biomedicine, Hangzhou, Zhejiang Province China; 3 Institute of Basic Medical Sciences, Westlake Institute for Advanced Study, Hangzhou, Zhejiang Province China; 4 Division of Cellular and Molecular Neurobiology, Zoological Institute, Technische Universität Braunschweig, Braunschweig, Germany; 5 College of Physics, Jilin University, Changchun, 130012 Jilin Province China; 6 Key Laboratory of Growth Regulation and Translational Research of Zhejiang Province, School of Life Sciences, Westlake University, Hangzhou, China

**Keywords:** Neuroscience, Biotechnology, Expression systems

## Abstract

Spectrally diverse fluorescent proteins (FPs) provide straightforward means for multiplexed imaging of biological systems. Among FPs fitting standard color channels, blue FPs (BFPs) are characterized by lower brightness compared to other spectral counterparts. Furthermore, available BFPs were not systematically characterized for imaging in cultured mammalian cells and common model organisms. Here we introduce a pair of new BFPs, named Electra1 and Electra2, developed through hierarchical screening in bacterial and mammalian cells using a novel dual-expression vector. We performed systematic benchmarking of Electras against state-of-art BFPs in cultured mammalian cells and demonstrated their utility as fluorescent tags for structural proteins. The Electras variants were validated for multicolor neuroimaging in *Caenorhabditis elegans*, zebrafish larvae, and mice in comparison with one of the best in the class BFP mTagBFP2 using one-photon and two-photon microscopy. The developed BFPs are suitable for multicolor imaging of cultured cells and model organisms in vivo. We believe that the described dual-expression vector has a great potential to be adopted by protein engineers for directed molecular evolution of FPs.

## Introduction

During the past few decades, there is an increasing need for simultaneous high-content imaging of multiple subcellular and cellular structures in intact biological systems. Spectral diversity of fluorescent proteins (FPs) provides a straightforward approach for multiplex imaging of different subcellular and cellular structures in cultured cells^1–4^ and various model organisms^5–8^. For example, investigation of clonal expansion and tissue plasticity as well as neuronal tracing are heavily reliant on spectral diversity of multicolor strategies^9,10^. One of the major criteria when selecting a genetically encoded FP for most in vivo applications is fluorescence brightness, among others^11^. According to FPbase (https://www.fpbase.org/)^12^ there are multiple available FPs with high molecular brightness, mostly emitting cyan, green, yellow, and red fluorescence. However, there is mounting evidence supported by numerous studies that high molecular brightness does not always correspond to high intracellular brightness in cultured cells and in vivo^13–16^. This introduces a need for testing newly developed FPs under various experimental conditions including expression vectors, cell types, model organisms etc*.*, in order to evaluate their performance and provide valuable insight for end-users.

In addition to intracellular brightness, the monomeric state of FPs is considered a prerequisite for many applications, mainly because monomeric FPs are advantageous over their dimeric or tetrameric counterparts since they allow for artifact-free protein-of-interest tagging. Currently available monomeric green^17^ and yellow^18^ FPs are characterized by the highest molecular brightness and are usually selected in combination with red^19^ and blue^20,21^ FPs; a combination that provides crosstalk-free spectral multiplexing conditions using standard one-photon^8,22^ and two-photon^23,24^ imaging setups. However, blue FPs^20,21,25^ exhibit significantly lower brightness compared to green, yellow, and red FPs. Furthermore, available blue FPs were not systematically characterized in culture mammalian cells and model organisms in vivo.

To address these limitations, we engineered two monomeric blue FPs (BFPs), named Electra1 and Electra2, with optimized intracellular brightness and conducted their benchmarking against the top-performing BFPs reported to date. To optimize intracellular brightness, we introduced a novel protein optimization approach using a hybrid expression system, which enabled a high-throughput assessment of intracellular brightness in bacterial and mammalian cells. Electra1 and Electra2 exhibited high performance as fluorescent tags for structural proteins in mammalian cells. We also demonstrated the utility of Electra1 and Eectra2 for multicolor neuroimaging in vivo in model organisms such as *Caenorhabditis elegans*, zebrafish, and mice in combination with conventional green and red FPs using one-photon and two-photon microscopy. We believe that the dual-expression vector and the presented method of hierarchical screening can be readily deployed for protein engineering of other novel FPs.

## Results

### Development of optimized BFPs using a hybrid expression vector

It was previously shown that GFP-like RFPs can be converted into bright BFPs by targeting amino acid positions in close proximity to the chromophore^21,25^. To engineer the next-generation BFPs, we utilize a monomeric bright RFP, mRuby3, as a template for the site-directed mutagenesis according to the previously described strategy^26^. Screening of the generated library revealed multiple clones with exclusive blue fluorescence. For further optimization of the identified variants, we decided to apply hierarchical screening of random libraries in bacterial and mammalian cells to select BFPs with enhanced intracellular brightness.

**Figure 1:**
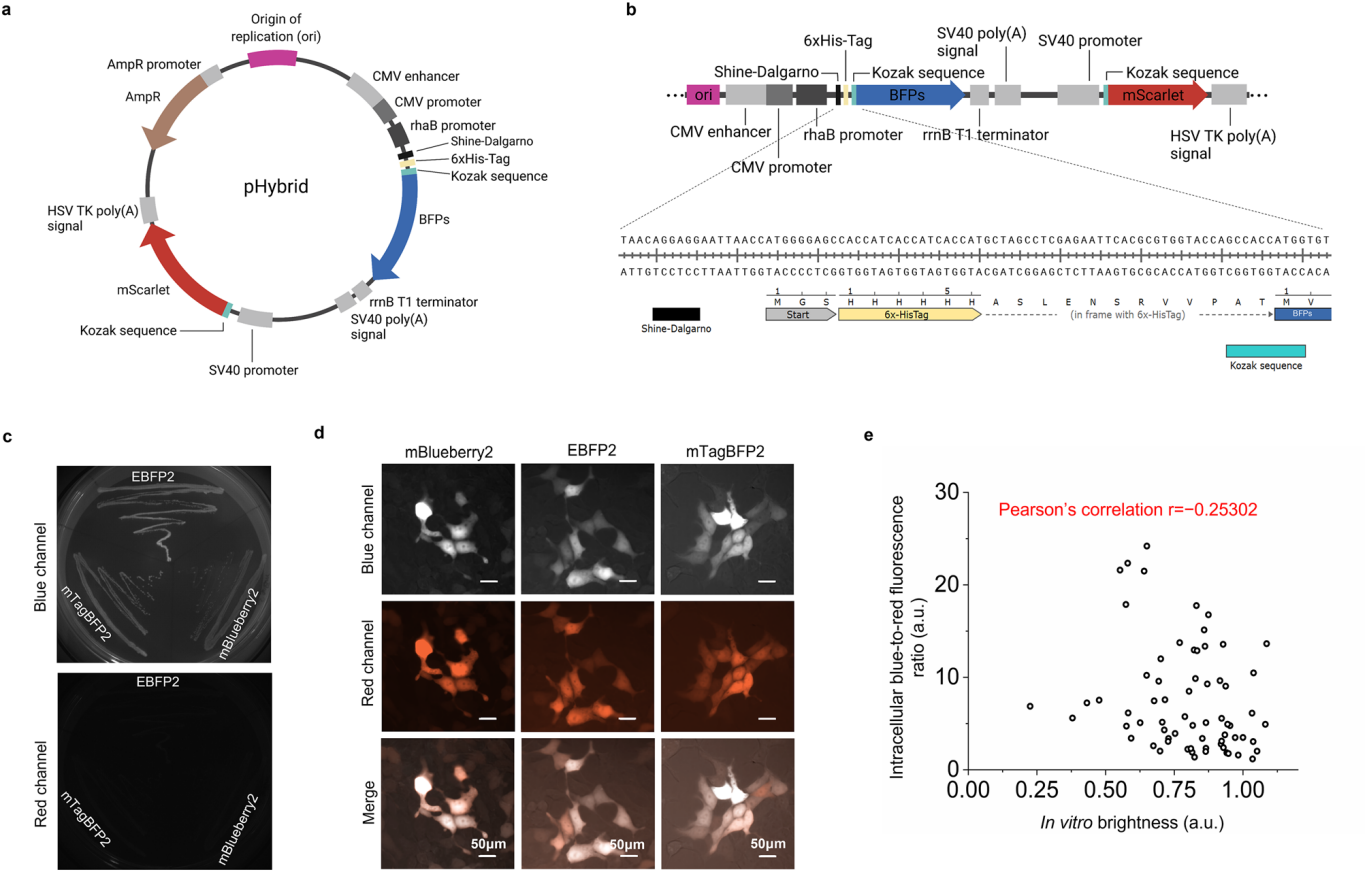
Development and validation of new pHybrid expression vector. (**a**) pHybrid-BFP-mScarlet vector map. Introduction of a new hybrid vector for bacterial and mammalian expression of target gene using mScarlet as reference gene. (**b**) pHybrid linear map showing basic features of pHybrid vector: (top) origin of replication, BFPs under CMV promoter and rhaB promoter for expression in mammalian cells and *E. coli*, respectively, followed by rrnB T1 terminator and SV40 poly-A tail, mScarlet under SV40 promoter regulation followed by HSV TK poly-A tail; (bottom) nucleotide sequence of the SD sequence, His-tag and Kozak sequence elements in front of the BFP gene. (**c**) Fluorescence images of *E. coli* bacteria transformed with pHybrid-mBlueberry2/mScarlet (mBlueberry2), pHybrid-EBFP2/mScarlet (EBFP2), and pHybrid-mTagBFP2/mScarlet (mTagBFP2) expression vectors in blue (top) and red (bottom) channels (imaging conditions: 403 nm/456 nm excitation/emission for blue, ex/em 561 mn/594 nm for mScarlet). (**d**) Fluorescence images of HEK cells transfected with pHybrid-mBlueberry2/mScarlet (mBlueberry2), pHybrid-EBFP2/mScarlet (EBFP2), and pHybrid-mTagBFP2/mScarlet (mTagBFP2) expression vectors in blue (top), red channel (middle) and merged (bottom) (imaging conditions: 403 nm excitation, 456 nm emission for BFPs; 561 nm excitation, 594 nm emission for mScarlet, 0.91 mW/mm^2^ power). The dynamic range of all images was adjusted independently to facilitate visualization. (**e**) Correlation of HEK intracellular brightness and in vitro brightness for blue fluorescence variants selected from the random library of BFP derived from mRuby3 (see Supplementary Table S1 for statistics; Pearson’s correlation shown in graph; imaging conditions of HEK same as in (**d)**).

To streamline optimization of intracellular brightness of FPs using directed molecular evolution, we aimed to append high-throughput screening of large gene libraries expressed in *E.coli* with a rapid assessment of intracellular brightness of selected clones in mammalian cells. An optimal expression vector for this goal would provide efficient expression of target genes in common bacterial strains and mammalian cells. In addition, expression in bacterial cells should be tightly regulated to prevent negative selection and protein expression-compatible bacterial strains should provide a sufficient yield of high-quality DNA that can be used for sequencing and direct transfection into mammalian cells. To meet these requirements, we designed a simple cloning plasmid system, denoted pHybrid, for gene expression in *E. coli* and mammalian cells. The pHybrid vector combines bacterial and mammalian expression cassettes comprising of CMV promoter, the L-rhamnose-inducible rhaB promoter, the Shine–Dalgarno (SD) sequence, bacterial rrnB T1 terminator, and SV40 poly-A tail (Fig. 1a, b). In addition, it contains independent expression cassette with SV40 promoter for mammalian expression of a reference FP to normalize for the overall expression level of target gene (Fig. 1a, b).

To validate the new pHybrid vector, we first expressed mBlueberry2^25^, EBFP2^21,25^, and mTagBFP2^20^ proteins under the CMV-rhaB hybrid promoter in TOP10 *E. coli* cells. Expression level of the target genes was sufficient for screening of colonies under fluorescence microscopy (Fig. 1c) and for protein extraction and purification using metal affinity chromatography. At the same time, expression of the reference gene, mScarlet, was not observed in *E. coli* cells (Fig. 1c). Plasmid DNA purified from TOP10 cells enabled efficient co-expression of the target and reference genes in HEK cells (Fig. 1d).

**Figure 2:**
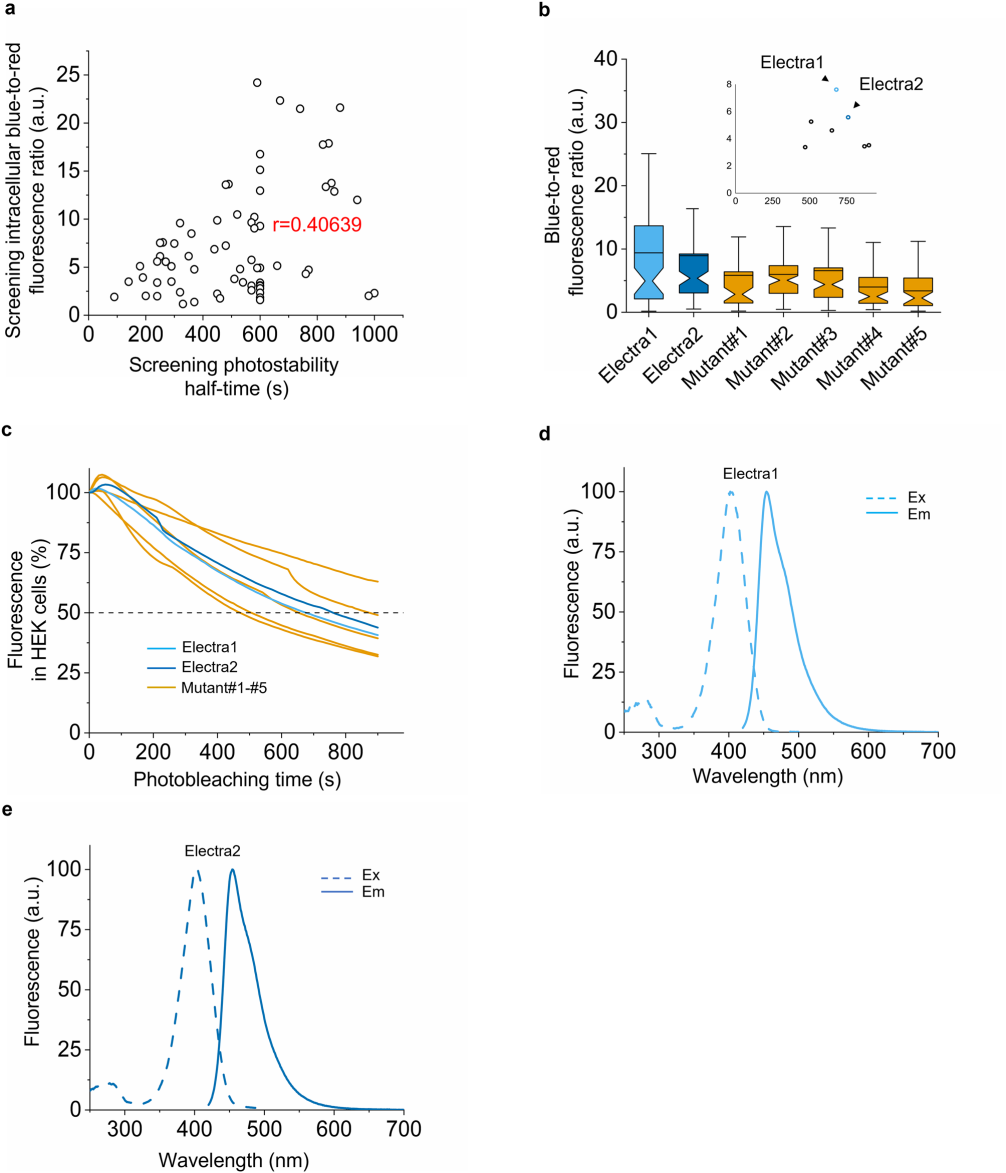
Screening of blue mRuby3 variants, in vitro characterization of two best performing mutants. (**a**) Intracellular brightness and photostability half-times of 67 variants expressed in HEK cells using pHybrid-/mScarlet expression under screening conditions. Imaging conditions for intracellular brightness of BFP variants: 403 nm LED excitation, 456 nm emission; for half-life measurement: 403 nm LED excitation, 456 nm emission (Supplementary Table S2). (**b**) Blue-to-red fluorescence ratio in live HEK cells expressing 7 best performing blue mRuby3 variants using pHybrid-/mScarlet (*n* = 73, 71, 48, 89, 80, 72, 55 cells from one transfection; Kruskal–Wallis ANOVA *p*-value = 8.4e-8; Supplementary Table S3; imaging conditions for BFPs: 403 nm LED excitation, 456 nm emission, 0.91 mW/mm^2^ power). Narrow part of notch, median; top and bottom of the notch, 95% confidence interval for the median; top and bottom horizontal lines, 25% and 75% percentiles of the data; whiskers extend 1.5 × IQR from the 25th and 75th percentiles; horizontal line, mean; dots, outliers. Subplot shows intracellular brightness (y’y) and photostability half-times (x’x) resulted from (**b, c)**. Electra1 is shown in light blue, Electra2 in dark blue throughout. (**c**) Time dependent intracellular fluorescence as part of the screening process in HEK cells using pHybrid-/mScarlet as expression vector (*n* = 10, 10, 10, 10, 11, 11, 11 cells from one transfection; imaging conditions for BFPs: 403 nm LED excitation, 456 nm emission; 4.25 mW/mm^2^ power). (**d, e**) In vitro excitation and emission spectra of Electra1 and Electra2, respectively. Excitation was measured in the range 250 nm-500 nm and emission from 420 to 700 nm for both purified proteins; Ex peak at 402 nm for Electra1; Ex peak at 403 nm for Electra2; Em peak at 456 nm for both proteins.

Having validated pHybrid vector for the expression of individual proteins, we tested it for high-throughput screening of large gene libraries. The pool of BFPs identified in the site-directed mutagenesis was subjected to error-prone PCR to generate random library consisting of about 10^7^ independent clones. The library was expressed in *E. coli* and subjected to screening with a fluorescence activated cell sorter (FACS) followed by selection of the blue fluorescence colonies on Petri dishes using a fluorescence stereomicroscope. About 100 selected clones were expressed in *E. coli* for in vitro measurements of spectral properties and brightness, and in HEK cells for assessment of intracellular brightness. Based on the in vitro brightness, the selected variants were ranked according to their normalized emission intensity (higher to lower) and correlated with normalized blue-to-red fluorescence ratio in HEK cells. We found that there was a low degree of negative correlation between emission in *E. coli* and intracellular brightness in HEK cells (Fig. 1e; see Supplementary Table S1 for full statistics), a behavior that can be attributed to lower folding efficiency in bacterial cells compared to HEK cells, as previously reported for GFP variants^27^. These results demonstrated the need for screening variants in mammalian cells for identifying those with improved intracellular brightness. Confirming that pHybrid system can be used for hierarchical screening in bacterial and mammalian cells, we performed two more rounds of directed evolution as described above, selecting about 10 best clones from each round for further evolution. For the final round of evolution, we additionally measured intracellular photostability in HEK cells, as photostability during live imaging is an important feature of FPs and a prerequisite for FP selection in many in vivo applications^11,28,29^. Pearson’s correlation of intracellular blue-to-red fluorescence ratio and photobleaching half-time demonstrated medium positive correlation (r = 0.40639, Fig. 2a, Supplementary Table S2). Among the 67 unique variants screened, we selected 7, based on the product of normalized brightness and photostability to confirm that the observed improvements were statistically significant. Two variants exhibited at least 30% higher score compared to the rest of the selected clones (Fig. 2b-c, Supplementary Table S3, see Supplementary Fig. S4 for photobleaching curves ± standard error of mean (SEM)). Sequencing of the 7 variants revealed the following common mutations K10E/M67L/Y68F/R71K/E118G/K142E/N147F/C176A/F178I/H201Y/Q217L with the following unique mutations for the two top-performing variants D155N/G171D/N190D/I206V and K85E/P131H/N133K/Y236F compared to the parental mRuby3 protein (numbering based on mRuby3 sequence; Supplementary Fig. S1 for amino acid alignment; Supplementary Fig. S2 for structures). The two selected variants were correspondingly named Electra1 and Electra2 (named after a water nymph in ancient Greek mythology). Electra1 and Electra2 had very similar fluorescence spectra with excitation maxima at 402 and 403 nm, respectively, and identical emission spectra with maximum at 456 nm (Fig. 2d-e).

### Characterization of Electra variants in cultured mammalian cells

**Figure 3:**
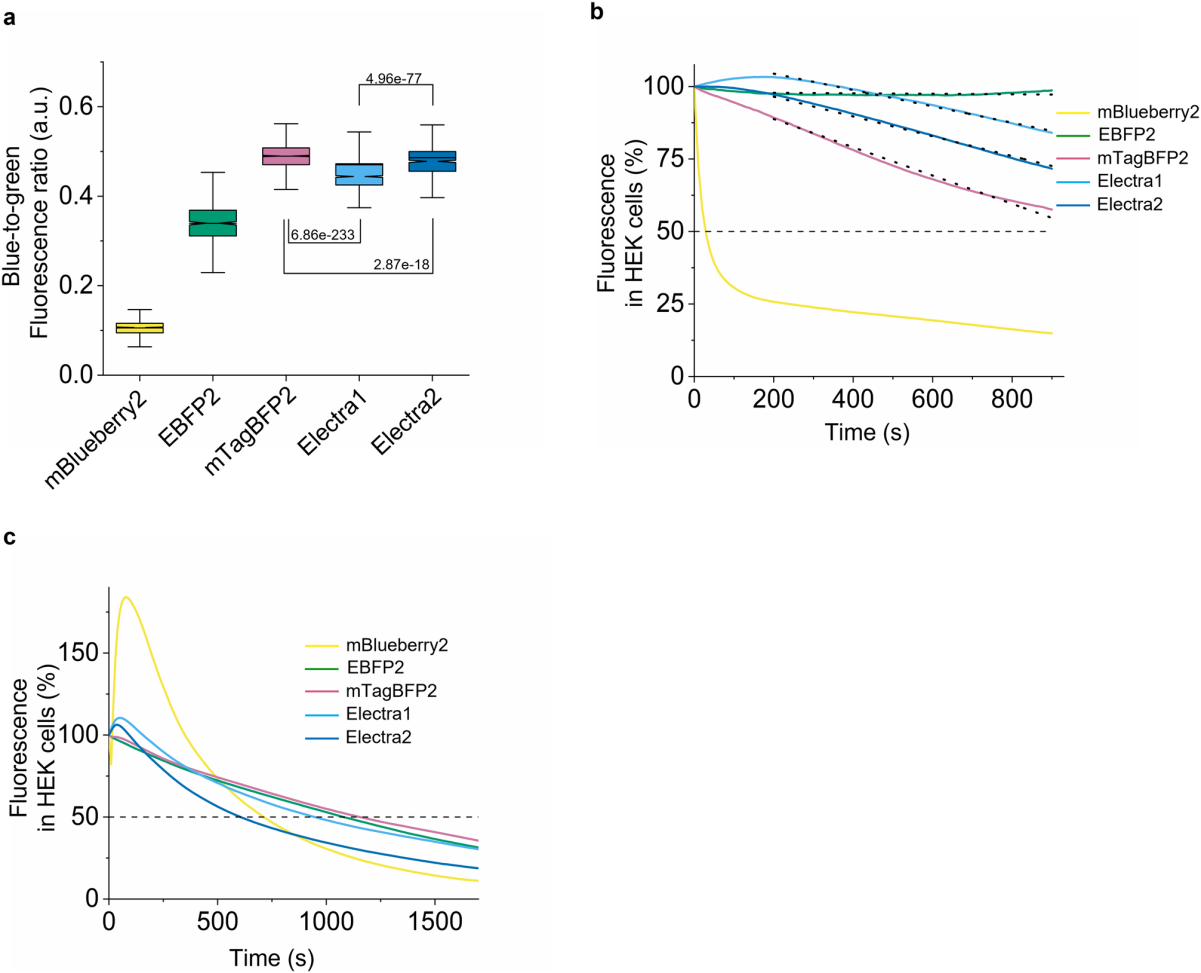
Characterization of Electra1, Electra2 in mammalian cell culture. (**a**) Blue-to-green fluorescence ratio in live HEK cells co-expressing mBlueberry2, EBFP2, mTagBFP2, Electra1, and Electra2 with EGFP via the P2A self-cleaving peptide (*n* = 835, 573, 1698, 1229, and 664 cells respectively, 4 independent transfections each; Kruskal–Wallis ANOVA, *p*-value = 0; post-hoc Kolmogorov-Smirnoff two sample *p*-values shown in graph; Supplementary Table S4). Imaging conditions for BFPs: 403 nm LED excitation, emission 456 nm, 0.91 mW/mm^2^ power. Box plots same as in Fig. 2b. (**b**) Normalized photobleaching curves for mBlueberry2, EBFP2, mTagBFP2, Electra1 and Electra2 expressed in HEK cells (*n* = 97, 81, 122, 105, 70 cells respectively, 4 independent transfections each; imaging conditions for BFPs: excitation 403 nm LED excitation, emission 456 nm, 0.91 mW/mm^2^ power). Dotted lines represent linear fit (decrease rate for EBFP2, mTagBFP2, Electra1, Electra2 in fluorescence %/sec: 8.33195e-4, 487.4e-4, 282e-4, 341.3e-4, respectively, fitting details in Supplementary Table S4). (**c)** Normalized photobleaching curves for mBlueberry2, EBFP2, mTagBFP2, Electra1 and Electra2 expressed in HEK cells (*n* = 63, 79, 137, 144, 90 respectively; 4 independent transfections each; imaging conditions for HEK cells: 403 nm LED excitation, 456 nm emission, 4.25 mW/mm^2^ power).

To benchmark performance of the Electra variants in cultured mammalian cells, we measured their intracellular brightness, photostability, and oligomeric state in comparison with mBlueberry2^25^, EBFP2^21,25^, and mTagBFP2^20^ as the best performing BFPs in terms of brightness and photostability reported to date. To correct for expression level variability, the selected BFPs were co-expressed with EGFP via P2A self-cleaving peptide and blue-to-green fluorescence ratios were used as a measure of intracellular brightness. When transiently expressed in HEK cells, Electras were more than fourfold brighter than mBlueberry2 and about 1.4-fold brighter than EBFP2, while slightly dimmer than mTagBFP2 (Fig. 3a, Table 1). All five proteins tested were evenly localized in cytoplasm of HEK cells with no visible aggregates (Supplementary Fig. S3). When we were assessing intracellular photostability in HEK cells, we observed that photobleaching rates were strongly dependent on illumination power. Therefore, to measure the intracellular photostability, we used two different illumination conditions: low excitation power (0.91 mW/mm^2^), which corresponds to live cell imaging conditions, and high excitation power (4.25 mW/mm^2^). According to the photostability at the lower light power, BFPs were ranked as following EBFP2 > Electra2 > Electra1 > mTagBFP2 > mBlueberry2, while at high power the ranking was strikingly different mTagBFP2 > EBFP2 > Electra1 > mBlueberry2 > Electra2 (Fig. 3b-c, Table 1, see Supplementary Fig. S4 for photobleaching curves ± SEM). These results demonstrate the need for setting up appropriate imaging conditions for live cell imaging using BFPs (Table 1). Since mBlueberry2 exhibited lower performance compared to other BFPs it was excluded from further benchmarking experiments.

**Table 1:** Spectral characteristics and performance of the selected BFPs.

	mBlueberry2	EBFP2	mTagBFP2	Electra1	Electra2
Ex max (nm)	402^25^	383^21^	399^20^	402	403
Em max (nm)	467^25^	448^21^	454^20^	456	456
Relative brightness in HEK cells^a^	21	69	100	91	97
Photostability in HEK cells (low power, s)^b^	25	57,500^c^	996^c^	2127^c^	1466^c^
Photostability in HEK cells (high power, s ± SEM)^b^	710 ± 70	1080 ± 25	1150 ± 45	950 ± 45	610 ± 50
Relative brightness in cultured neurons^a^	N.D	70	100	78	91
Photostability in neurons (s ± SEM)^b^	N.D	635 ± 70	135 ± 5	185 ± 10	200 ± 5
Relative brightness in *C. elegans*^a^	N.D	N.D	100	237	218
Relative brightness in zebrafish (hb)^a^	N.D	N.D	100	82	117
Relative brightness in zebrafish (sc)^a^	N.D	N.D	100	95	110
Photostability in zebrafish (sc, s ± SEM)^b^	N.D	N.D	39 ± 2.2	28.6 ± 2.6	20.8 ± 1.6
Relative brightness 2photon^a^	N.D	N.D	100	121	87
OSER assay^d^	N.D	57 ± 2.7^31^ 36.3^12^	49.8 ± 1.9^31^ 68.9 ± 3.3^e^	65.6 ± 4.6^e^	61.2 ± 1.8^e^
MFI:NE MFI ± SEM	N.D	2.6 ± 0.08	2.9 ± 0.1	2.7 ± 0.1	2.7 ± 0.09

^a^Refers to mean intracellular brightness ratio normalized to mTagBFP2;

^b^Refers to mean fluorescence half-time;

^c^Estimated using linear fit extrapolation;

^d^Fraction of cells with normal ER reported as % of cells ± SEM;

^e^Values reported in this study.

**Figure 4:**
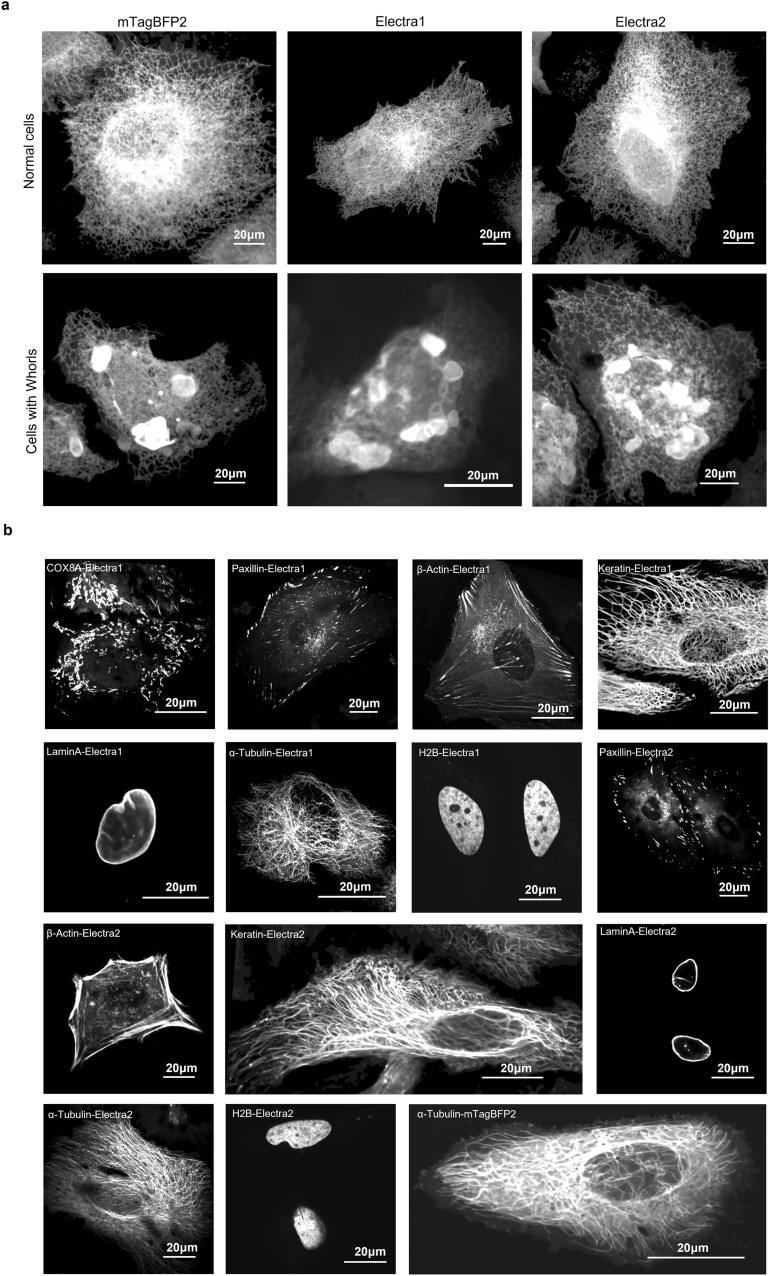
Electra variants performance as fusion tags in live HeLa cells. (**a**) -terminal fusion of mTagBFP2, Electra1 and Electra2 with CytERM for OSER assay (one transfection each; see Supplementary Table S5). Representative images of healthy cells and cells with whorls are shown (403 nm excitation; 430–490 nm emission). The dynamic range of all images was adjusted independently to facilitate visualization. (**b**) From top left to right: COX8A-Electra1, paxillin-Electra1, β-actin-Electra1, keratin-Electra1, laminA-Electra1, α-tubulin-Electra1, H2B-Electra1, paxillin-Electra2, β-actin-Electra2, keratin-Electra2, laminA-Electra2, α-tubulin-Electra2, H2B-Electra2, α-tubulin-mTagBFP2 (imaging conditions: 403 nm excitation; 490 nm emission). The dynamic range of all images was adjusted independently to facilitate visualization.

To characterize oligomeric tendencies of Electras in mammalian cells, we conducted OSER assay (Organized Smooth ER assay)^30,31^ using mTagBFP2 for a reference. Fractions of transfected HeLa cells with normal ER were 68.9 ± 3.3%, 65.6 ± 4.6%, and 61.2 ± 1.8% (mean ± SEM) for mTagBFP2, Electra1, and Electra2, respectively (Fig. 4a, Supplementary Table S5). In addition to fraction of cells with normal ER, Costantini et al. proposed a quantitative comparison of ratios of whorl structure mean fluorescence intensities (MFI) to nuclear envelope (NE) MFI as a way to further validate true monomeric behavior^30^. To this end we assessed EBFP2, mTagBFP2, Electra1 and Electra2, which resulted in ratios of 2.6 ± 0.08, 2.9 ± 0.1, 2.7 ± 0.1, 2.7 ± 0.09 (mean ± SEM), respectively, a slightly different ranking compared to the fraction of normal cells (Table 1, Supplementary Table S5). Although both OSER assays can provide valuable insight into the oligomerization tendency of FPs in mammalian cells, we observed high variability among experiments in MFI ratios and fraction of cells from previous studies^31,32^ which can be attributed to expression level stochasticity, post-transfection incubation time, and imaging conditions. We advise these numbers to be used with caution and avoid comparison with future values unless a side-by-side assessment under identical conditions is implemented. According to Cranfill et al*.*^31^, FPs with OSER scores between 41 and 68% should be tested independently in demanding fusions, such as structural proteins, to ensure their correct function and localization. Therefore, the next step was to evaluate the performance of Electras as protein fusion tags for structural proteins (Fig. 4b). We tested the following fusions in HeLa cells: partial cytochrome C oxidase subunit VIII (COX8A), paxillin, β-actin, keratin, laminA, α-tubulin, and H2B. mTagBFP2 was used as a reference for fusion tag performance with α-tubulin. As expected from OSER assay results, both Electra variants performed well in all tested fusions except for COX8A-Electra2, which exhibited significant cytoplasmic localization (Supplementary Fig. S5).

**Figure 5:**
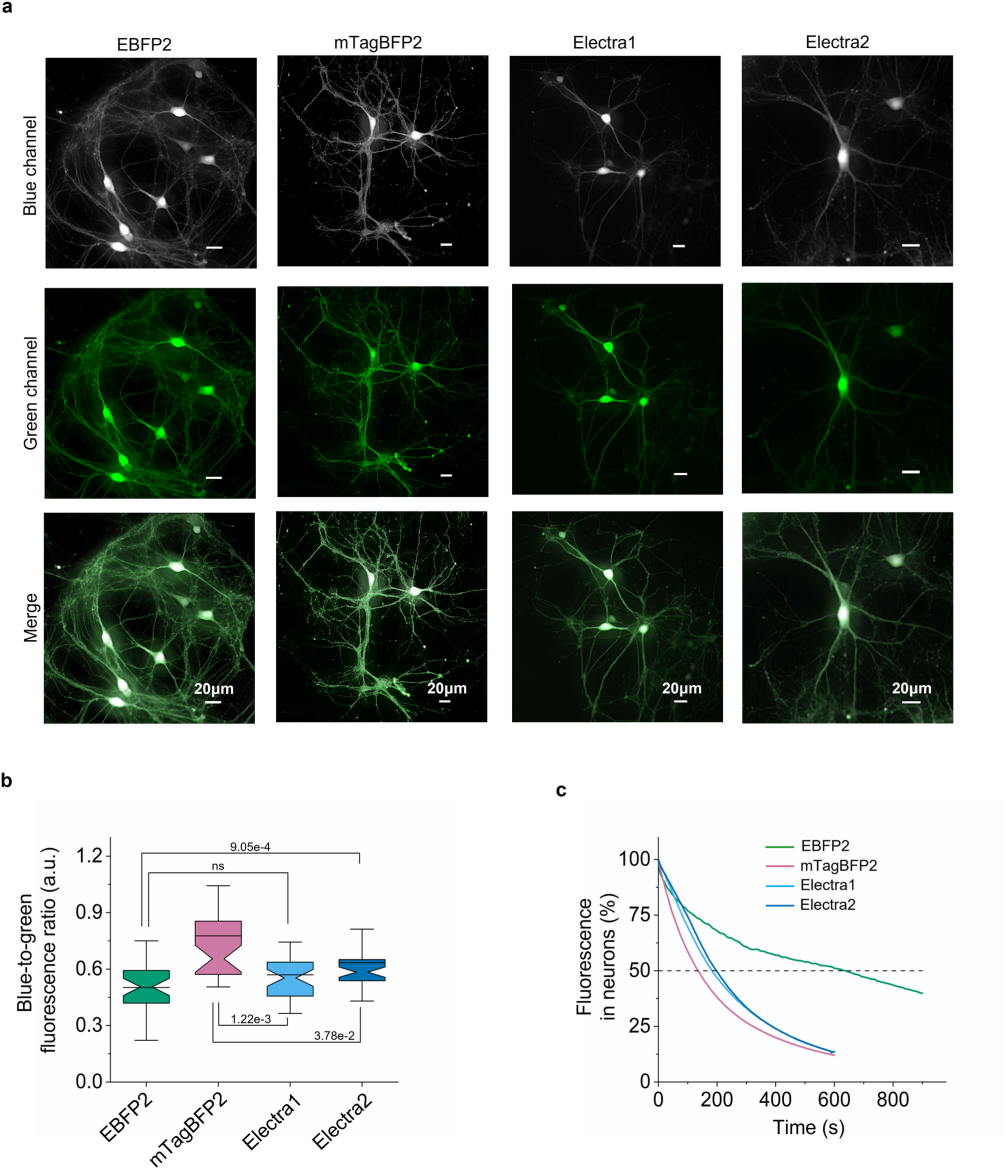
Characterization of Electra variants in cultured neurons. (**a**) Representative images of hippocampal neurons at DIV14 co-expressing EBFP2, mTagBFP2, Electra1, or Electra2 under human synapsin promoter with EGFP via P2A self-cleaving peptide. BFPs are shown in grey; EGFP in green (*n* = 35, 37, 38, 40 respectively, from 2 independent rAAV2/9 transductions each; imaging conditions for BFPs: 403 nm excitation; 456 nm emission; 0.91 mW/mm^2^; z-stack max projection; image deconvolution using NIS Elements online deconvolution tool). The dynamic range of all images was adjusted independently to facilitate visualization. **(b**) Quantification of blue-to-green fluorescence ratio in live hippocampal neurons (*n* = 35, 37, 38, 40 neurons for EBFP2, mTagBFP2, Electra1, Electra2, respectively, from 2 independent transductions each; Kruskal–*WaNllis* ANOVA *p*-value = 4.66e-8; post-hoc two sample Kolmogorov-Smirnoff *p*-values shown in graph; Supplementary Table S6). Box plots throughout same as in Fig. 2b. (**c**) Photobleaching curves for EBFP2, mTagBFP2, Electra1, Electra2 in live neurons under continuous wide-field illumination (*n* = 8, 26, 32, and 26 neurons, respectively; imaging conditions of BFPs: continuous illumination under 403 nm LED, 3.72 mW/mm^2^).

We continued the benchmarking of the selected BFPs in the cytosol of primary hippocampal mouse neurons through transduction with rAAV2/9 under human synapsin promoter. All tested BFPs demonstrated even cytoplasmic labeling that enabled visualization of both somas and dendrites of hippocampal neurons (Fig. 5a). The intracellular brightness of Electra1 and Electra2 was 1.28- and 1.1-fold lower than that of mTagBFP2, but 1.12- and 1.3-fold higher than that of EBFP2 (Fig. 5b, Table 1). In turn, EBFP2 was characterized by the highest photostability with photobleaching half-time of 635 ± 70 s followed by Electra1 and Electra2 with photobleaching half-time of 185 ± 10 s and 200 ± 5 s, respectively. mTagBFP2 demonstrated the lowest photostability with 135 ± 5 s photobleaching half-time, which was 1.4- and 1.5-fold lower than Electra1 and Electra2, respectively (Fig. 5c, Table 1, see Supplementary Fig. S4 for photobleaching curves ± SEM). Overall, performance of Electras in cultured mammalian cells in terms of intracellular brightness, photostability, and oligomeric state is comparable to that of mTagBFP2 with Electras being more suitable for live-cell imaging under low light power due to the higher photostability.

### Validation of the electra variants for neuroimaging in model organisms

**Figure 6:**
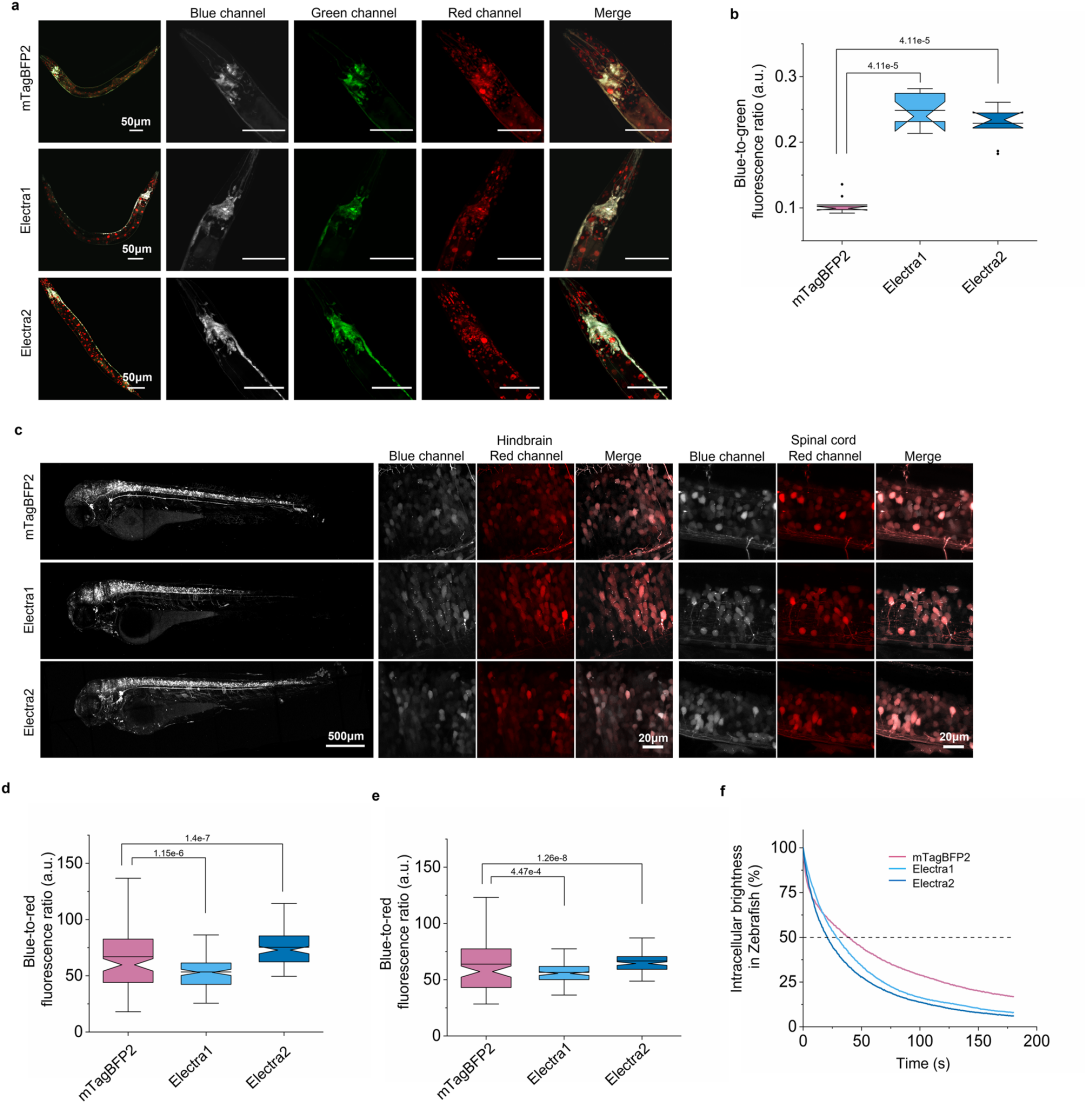
Fluorescence comparison of mTagBFP2, Electra1 and Electra2 in small model organisms. (**a**) Whole body images of representative *C. elegans* worms co-expressing mTagBFP2, Electra1 and Electra2 with mNeonGreen under pan-neuronal promoter *tag168* and mScarlet in most somatic cells. Higher magnification images of neuronal ring under blue (shown in grey), green (mNeonGreen), red channel (mScarlet) and merged. Imaging conditions: 405 nm excitation for BFPs, 430–470 nm emission; 488 nm excitation for mNeonGreen, 500–540 nm emission; 561 nm excitation for mScarlet, 570–620 nm emission. The dynamic range of all images was adjusted independently to facilitate visualization. (**b**) Blue-to-green fluorescence ratio comparison in *C. elegans* neuronal ring (*n* = 9 worms for each protein; Kruskal Wallis ANOVA *p*-value = 1.29e-4; post-hoc two sample Kolmogorov-Smirnoff *p*-values shown in graph; Supplementary Table S7). (**c**) Representative whole overview image of 4dpf zebrafish expressing each BFP throughout brain and spinal cord (left). Higher magnified images of blue, red, and merged fluorescence in hindbrain (middle) and spinal cord (right) neurons. Transient co-expression of each BFP and mScarlet was induced by injection into zebrafish embryos with a construct carrying each BFP-P2A-mScarlet whose expression is under the control of pan-neuronal *nbt* promoter. Imaging conditions: Blue channel: excitation 405 nm laser, emission 420–480 nm; red channel: excitation 561 nm laser, emission 565–606 nm). The dynamic range of all images was adjusted independently to facilitate visualization. (**d**) Blue-to-red fluorescence ratio comparison in zebrafish hindbrain expressing mTagBFP2, Electra1, Electra2 (*n* = 116, 117, 116 neurons from 4 independent zebrafish; Kruskal–Wallis ANOVA *p*-value = 1.98e-19; post-hoc two sample Kolmogorov-Smirnoff *p*-values shown in graph; Supplementary Table S8; imaging conditions for BFPs: same as in (**a)**). (**e**) Blue-to-red fluorescence ratio comparison in zebrafish spinal cord neurons expressing mTagBFP2, Electra1, Electra2 (*n* = 113, 118, 117 neurons from 4 independent zebrafish; Kruskal–Wallis ANOVA *p*-value = 1,2e-8; post-hoc two sample Kolmogorov-Smirnoff *p*-values shown in graph; Supplementary Table S8) with mScarlet. Imaging conditions for BFPs: same as in (**c**). (**f**)Time-dependent blue fluorescence measurement for mTagBFP2, Electra1, Electra2 (*n* = 40, 40, 40, respectively from 4 independent zebrafish) in spinal cord neurons. Imaging conditions of BFPs: Blue channel: excitation 405 nm laser, emission 420–464 nm.

Multicolor barcoding of neurons via stochastic FPs expression enables a powerful approach for mapping and tracing brain circuits^10^. To explore the utility of Electras for neuroimaging, we performed multicolor imaging of model organisms including *C. elegans*, zebrafish larvae, and mice under one- and two-photon microscopy. As a reference protein, we used mTagBFP2, which demonstrated superior intercellular brightness in cultured neurons. First, we co-expressed the selected BFPs with mNeonGreen in neurons and mScarlet in nuclei of somatic cells of *C. elegans*. Both Electras enabled cross-talk-free multi-color imaging using standard blue, green, and red channels, however, in the case of all three BFPs we observed formation of bright puncta in neurons, which was less significant for mTagBFP2 and Electra2, but slightly more significant for Electra1 (Fig. 6a). However, it should be noted that the puncta were also visible in the green channel, co-localizing with those visible in the blue channel. Quantification of intracellular brightness revealed that Electra1 and Electra2 were 2.3- and 2.1-fold brighter than mTagBFP2 (Fig. 6b).

Next, the Electra variants and mTagBFP2 were transiently co-expressed with mScarlet under pan-neuronal promoter in zebrafish larvae and visualized at 4 days post fertilization (Fig. 6c). Using high-resolution confocal imaging of neurons, we observed that Electra1 formed bright aggregates in neurons both in hindbrain and spinal cord, whereas mTagBFP2 and Electra2 are evenly distributed in neuronal cytoplasm throughout the larvae (Fig. 6c). Fluorescence brightness comparison revealed that Electra2 was 1.16- and 1.08-fold brighter than mTagBFP2 in hindbrain and spinal cord neurons, respectively, while Electra1 was slightly dimmer than mTagBFP2 in both neurons (Fig. 6d-e). To compare fluorescence photostability, we conducted continuous imaging of neurons in the spinal cord region. According to our results, mTagBFP2 outperforms Electras showing 1.38- and 1.87-fold higher photostability than Electra1 and Electra2, respectively (Fig. 6f, Table 1, see Supplementary Fig. S4 for photobleaching curves ± SEM).

**Figure 7:**
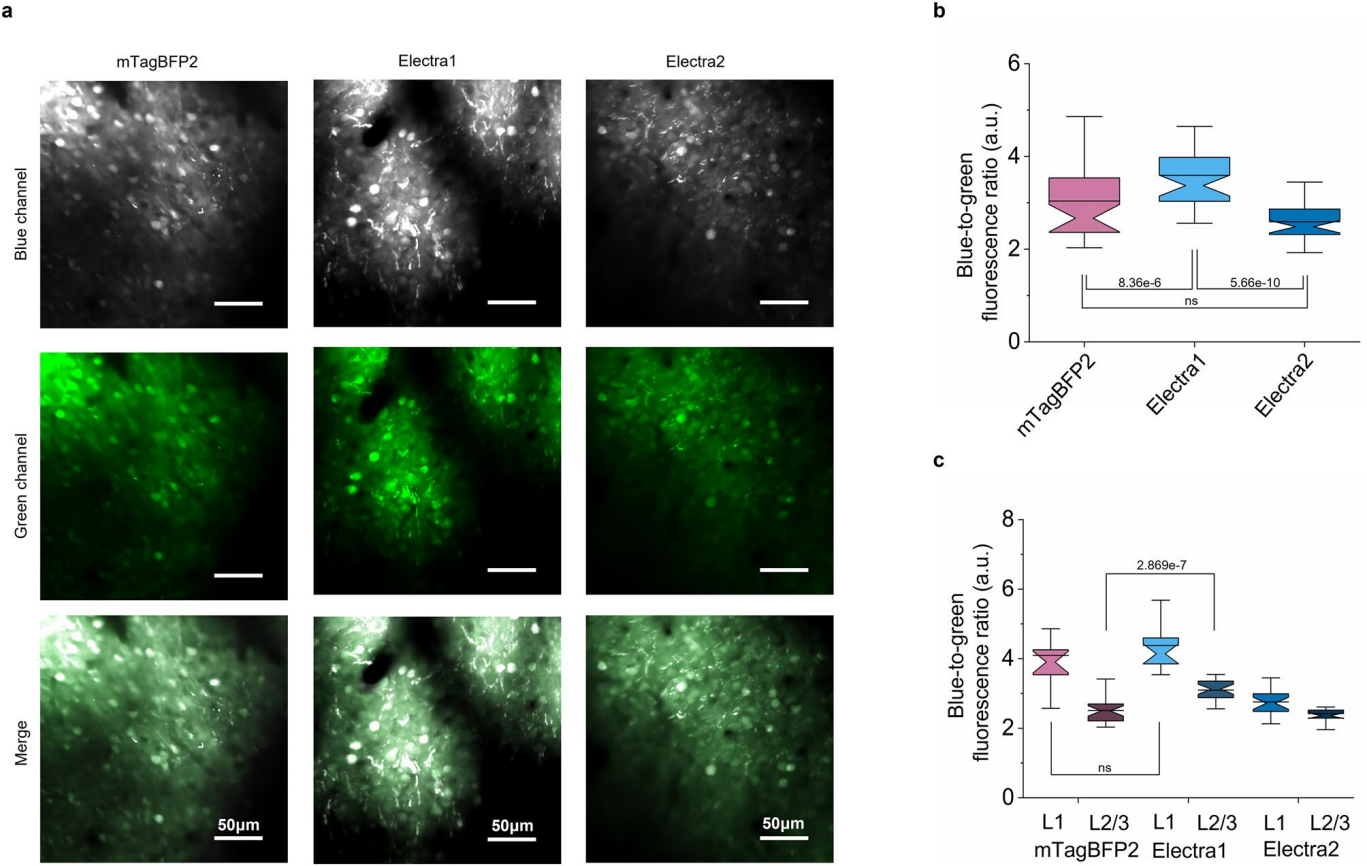
Comparison of EBFP2, mTagBFP2, Electra1, Electra2 in mouse cortex in vivo. (**a**) In vivo two-photon microscopy of cortex neurons co-expressing mTagBFP2, Electra1, or Electra2 with EGFP in live mice. Representative images of mTagBFP2, Electra1, Electra2 from cortex layer2/3 (~ 350 μm depth). The dynamic range was adjusted independently to facilitate visualization. (**b**) Blue-to-green fluorescence comparison in live mouse cortex neurons (L1 and L2/3) at P21 after injection expressing mTagBFP2, Electra1, Electra2 (*n* = 38, 43, 38 neurons, respectively, from one mouse each; Kruskal–Wallis ANOVA *p*-value = 9.63e-10, post-hoc Kolmogorov-Smirnoff *p*-values shown in graph; Supplementary Table S9). (**c**) Blue-to-green fluorescence ratio comparison in live mouse cortex presented independently for L1 and L2/3 (n_L1_ = 12, 16, 21 neurons for mTagBFP2, Electra1 and ELectra2, respectively; Kruskal–Wallis ANOVA *p*-value_L1_ = 7.7e-8; post-hoc KS *p*-values shown in graph; n_L2/3_ = 26, 27, 17 neurons for mTagBFP2, ELectra1 and ELectra2, respectively; KW ANOVA *p*-value_L2/3_ = 1.04e-9; post-hoc KS *p*-values shown in graph; Supplementary Table S9).

To test the Electra variants for neuroimaging in mice, we expressed them in the cortex of mouse brain by injecting the rAAV2/9-Syn-BFPs-P2A-EGFP virus at the neonatal stage and imaged them in four-week old mice under two-photon microscopy (Fig. 7a, Supplementary Fig. S6 for representative images of BFPs across depths). Similar to that observed in primary hippocampal mouse neurons, both Electra variants showed even cytoplasmic labeling without signs of aggregation or mislocalization. Quantification of the blue-to-green fluorescence ratio of neuronal somas revealed that intracellular brightness of Electra1 was 1.2-fold and 1.4-fold higher compared to mTagBFP2 and Electra2, respectively; the difference of mTagBFP2 and Electra2 brightness was not statistically significant (Fig. 7b). We further compared the brightness of BFPs in deeper cortex layers of neuronal somas from layer 1 and 2/3 independently. Electra1 showed significantly higher baseline brightness in layer 2/3 compared to mTagBFP2 from the same depth whereas there was no significant difference in layer1 (Fig. 7c). Histological analysis confirmed expression in the target brain regions (Supplementary Fig. S7). Using high-resolution imaging of the fixed brain sections, we observed bright blue fluorescence puncta in neurons expressing Electra1 and some minor aggregation in TagBFP2-expressing neurons. For comparison, EBFP2 and Electra2 exhibited even localization in cell bodies in processes (Supplementary Fig. S7). Altogether, our results demonstrated the applicability of Electras for multicolor neuroimaging in model organisms with similar performance compared to mTagBFP2. However, end-users should consider the tendency of Electra1 to form bright puncta upon expression in neurons in vivo.

## Discussion

In this study, we developed two new BFPs, named Electra1 and Electra2, characterized by intracellular brightness and photostability suitable for multicolor neuroimaging in combination with other conventional FPs. To optimize intracellular brightness in mammalian cells, we coupled the speed and convenience of high-throughput screening of large bacterial libraries with high-content mammalian cell-based screening for intracellular brightness and photostability without a need for the re-cloning step. This was achieved through the development of a dual-expression system, named pHybrid, by combining bacterial-specific and mammalian-specific sequences for efficient protein expression and purification both in *E.coli* and mammalian cells. The pHybrid has several unique advantages in comparison with previously developed dual-expression systems, such as pDRESS^33^ and pDual-GC (Agilent Technologies). For example, in contrast to pDRESS vector, which utilizes similar mammalian and bacterial promoters for driving target gene expression, pHybrid can be used with commercially available TOP10 strain and has an independent expression cassette for the reference gene thus enabling purification of the target gene for spectroscopic characterization without contamination from the reference protein. In addition, the rhamnose promoter enables tighter gene expression regulation compared to T7 promoter used in the pDual-GC vector. The introduced screening approach was successfully used for the development and optimization of BFP variants in only two rounds of directed molecular evolution starting from the bright RFP mRuby3. We believe that the pHybrid plasmid can find wide application as a dual-expression vector and the presented method of hierarchical screening can be adopted for protein engineering.

Quantitative assessment of Electra1 and Electra2 in mammalian cells revealed non-linear dependence of photobleaching rate on the illumination intensity. Similar behavior was observed for the other state-of-art BFPs, mBlueberry2, EBFP2, and mTagBFP2, used as references in this study. The mBlueberry2, mTagBFP2, and Electras proteins share the TagBFP-like chromophore, while EBFP2 has a GFP-like chromophore with a histidine ring^34,35^. Probably the higher photostability of EBFP2 under low-intensity illumination is due to its His-based chromophore. Both Electra1 and Electra2 exhibited minor photoactivation under transient transfection in HEK cells (Fig. 3b, c), which probably can be explained by photoconversion of non-fluorescent intermediate into the blue chromophore at a higher rate than photobleaching. Chromophore formation in BFPs derived from GFP-like RFPs via non-fluorescent chromophore precursor is a rate-limiting step^34^, which can be accelerated by UV illumination^36^. We suggest that upon transient expression in HEK cells (36 h) when the protein is actively translated there is a larger fraction of the C-form compared to that under long-term expression in cultured neurons (7 days) and zebrafish larva (4–5 days), where photoactivation was not observed. However, the exact molecular mechanism of this dependence remains unclear and may require further investigation, which is out of the scope of the current study. From the practical considerations, this property creates a need for careful selection of imaging conditions specifically when long-term imaging is the goal.

The OSER assay is a more accessible method for characterization of monomeric state of FPs in comparison to other well-established analytical methods, such as size-exclusion chromatography^16,37^ and analytical centrifugation^38^. However, in contrast to analytical methods, the OSER assay may not be suitable for the absolute quantification of monomeric state. For example, the values for EBFP2 reported in two independent studies showed significant differences, *i.e.*, 57% ± 2.7%^31^ and 36.3%^12^. The original study classified mTagBFP2 as a monomer^20^ and the subsequent study reported a 49.8% ± 1.9% fraction of normal cells^31^. The values for OSER assay reported for TagBFP2 in this study did not match well with previously reported number (Table 1). This discrepancy in the reported numbers can be due to variability of expression levels of CytERM-FP fusions in transiently transfected cells, which in turn may have a large effect on the appearance and morphology of the ER regardless of the FP involved. This is indicative of the OSER assays’ limitations regarding its universal application. Therefore, we performed a careful side-by-side comparison of the Electra variants with EBFP2 and mTagBFP2, which demonstrated similar monomericity of these BFPs in cultured cells. The monomeric nature of Electras was further confirmed by testing them in difficult fusions in mammalian cells. Overall, Electras exhibited similar performance in live cultured cells compared to the most widely used BFPs.

Although BFPs found a wide range of applications^39–43^, they have not been systematically tested for neuroimaging. In the present study, we evaluated the performance of novel BFPs for multicolor neuroimaging in vivo in three model organisms in comparison with mTagBFP2. Quantification of brightness revealed that in vivo brightness did not correlate with intracellular brightness measured in cultured cells. Moreover, in vivo brightness between species also was not consistent. In *C. elegans*, the Electra variants outperformed mTagBFP2 in terms of intracellular brightness by about two-fold. However, when comparing results from zebrafish expression, Electra1 had comparable brightness with mTagBFP2, but was dimmer than Electra2. Furthermore, while Electra2 outperformed mTagBFP2 and Electra1 in brightness in zebrafish, it was dimmer than Electra1 in *C. elegans*. Testing their performance using two-photon excitation in vivo in mouse brain we observed that Electra1 was 20% brighter than mTagBFP2 and about 40% brighter than Electra2. Similarly, the discrepancy in brightness between different preparation has been reported for several FPs^13,14,16,44–46^ demonstrating the need for validation of newly developed FPs in multiple expression systems depending on the intended use.

We also should note the higher tendency of Electra1 to form puncta in neurons in vivo. The GFP-like proteins, especially derived from DsRed, were previously reported to have a propensity to form brightly fluorescent puncta in neurons particularly during long term-expression^47^. The puncta were shown to co-localize with lysosomes but certain FPs due to the resistance to pH and lysosomal enzymes can retain their fluorescence^48,49^. It was also reported that freely diffusing mTagBFP2 formed visible aggregates in the hypodermis of *C. elegans,* though the exact mechanism was not investigated^50^. In our hands, all tested BFPs showed minor aggregation in cytoplasm of neurons in *C. elegans*. Additionally, when imaged in neurons fixed brain slices but not in vivo or cultured neurons, Electra1 and mTagBFP2 formed brightly fluorescent puncta, while EBFP2 and Electra2 did not produce fluorescent aggregates under the same conditions (Supplementary Fig. S7). These findings strongly support the differential behaviors of FPs depending on the expression system and demand rigorous and detailed observations under implemented experimental conditions. Although for structural imaging of neuronal morphology might not be significantly compromised, potential FP aggregation should be considered during protein selection for a given experiment.

Two rounds of directed molecular evolution were sufficient to develop novel BFPs, which exhibited performance comparable to that of the most widely used BFPs. Further optimization of Electras can involve enhancement of photostability and chemical stability of the chromophore. It was revealed that hydrolysis of the N-acylimine group in TagBFP is a major process contributing to chromophore degradation^20^. Two rational design strategies can be considered for improving chromophore stability in Electras. Mutagenesis of amino acid residues in the proximity to the N-acylimine group, such as Q43, M45, and S70 (Supplementary Fig. S2), is one approach to prevent its hydrolysis of the chromophore. The second approach can be based on optimization of chromophore forming tripeptide to explore, for example, if His-based chromophore, similar to that in EBFP2, results in higher photostability. It also should be noted that other enhanced RFPs, mScarlet, FusionRed, etc., are also promising templates for development of new BFPs.

## Materials and methods

### Molecular cloning, mutagenesis, and screening

The mBlueberry2 gene was synthesized de novo by Tsingke Biotechnology Co., Ltd. based on the sequence reported in the original publication^25^. Plasmids encoding mScarlet, EBFP2, and mTagBFP2 were acquired from Addgene. Synthetic DNA oligonucleotides used for cloning were purchased from Tsingke Biotechnology Co., Ltd. PrimeStar Max master mix (Clontech) was used for high-fidelity PCR amplifications. Restriction endonucleases were purchased from New England BioLabs and used according to the manufacturer’s protocols. Ligations were performed using T4 DNA ligase (New England BioLabs) or NovoRec® plus one step Cloning kit (Novoprotein). Small-scale isolation of plasmid DNA was performed with Mini-Prep kits (Qiagen); large-scale DNA plasmid purification was done with GenElute HP Endotoxin-Free Plasmid Maxiprep Kits (Sigma-Aldrich). Sequencing of purified plasmids and bacterial colonies was performed by Sanger method (Healthy Creatures Hangzhou, China).

The pHybrid plasmid was assembled in two steps using the pCI vector (Promega) as backbone. First, the rhamnose-induced expression cassette was PCR amplified from the pWA23h plasmid^51^ and inserted between CMV promoter and SV40 poly(A) sequence. Then, the SV40 expression cassette, consisting of SV40 promoter, mScarlet gene and HSV TK poly(A) tail, was cloned behind SV40 poly(A) sequence. pHybrid is high-copy number plasmid with the origin of replication of the filamentous phage f1 and ampicillin resistance. Target genes of BFPs with 6x-HisTag and short amino acid linker were cloned behind Shine-Dalgarno sequence for efficient bacterial translation^52^ and purification using metal affinity chromatography.

The mBlueberry2, EBFP2, mTagBFP2, Electra1, and Electra2 genes were PCR amplified as KpnI/AgeI fragment and swapped with the miRFP2 gene in the pAAV-CAG-miRFP2-P2A-EGFP plasmid^16^. For all fusions of Electra1 and Electra2 with structural proteins, pHybrid was used as template to amplify the target genes using primers with corresponding recombination sites for each construct. Cloning was performed by Tsingke Biotechnology Co. Ltd (China) using following plasmids as templates: pKeratin-miRFP670nano (Addgene plasmid #127437); pMito-iRFP713 (Addgene plasmid #45465); pPaxillin-TagRFP675-N1 (Addgene plasmid #44276); pmRuby2-Actin-7 (Addgene plasmid #55888); pTubuliln-miRFP670nano (Addgene plasmid #127429); pEBFP2-H2B-6-N1 (Addgene plasmid #55243); pmRuby2-LaminA (Addgene plasmid #55901); pCytERM-mScarlet-N1 (Addgene plasmid #85066).

Site-directed libraries of mRuby3 were synthesized de novo as gBlocks (EpochLifescience) with degenerated codons at the following amino acid positions 67, 68, 71, 84, 147, 162, 176, 178, 201. The gBlock was amplified with corresponding primers using PCR and subcloned into the pHybrid vector. Random mutagenesis was performed with GeneMorph II Random Mutagenesis Kits (Stratagene) under conditions enabling a mutation frequency of up to 15 mutations per 1000 base pairs. The generated gene libraries in expression vectors were electroporated into TOP10 *E. coli* host cells (Biomed). Serial dilutions (10^−4^ and 10^−5^) of the electroporated cells were plated on LB/agar medium supplemented with ampicillin to estimate electroporation and cloning efficiency. For each library 20 randomly selected clones were sequenced to estimate fraction of the clones containing target genes and mutation rate. The remainder of the cells was grown overnight in LB medium supplemented with ampicillin.

Typical random libraries for each screen with fluorescence-activated cell sorter (FACS) consisted of about 10^6^–10^7^ independent clones. To express generated libraries, TOP10 cells were grown at 37 °C overnight in LB with ampicillin 0.02% w/v L-rhamnose (Sangon Biotech (Shanghai) Co., Ltd.). Before FACS, the expressing bacteria were washed with phosphate-buffered saline (PBS) and then diluted with PBS to an optical density of 0.02 at 600 nm. FACS screening was performed with a BD FACSMelody cell sorter (BD Biosciences) blue channel (excitation 405 nm, emission 448/45 nm). About 10 sizes of each library were sorted for bacterial cells with the brightest blue fluorescence. The collected cells were rescued in rich SOC medium at 37 °C for one hour, and then plated on LB agar plates supplemented with Amp^+^ (0.1 mg/ml) and L-rhamnose (0.02%w/v). Colonies were analyzed under Olympus SZX16 stereomicroscope equipped with SPECTRA III light engine (Lumencor) and a color CCD camera (BGIMAGING). Colonies with the highest fluorescence in the blue channel (excitation 390/22 nm; emission 420 nm long pass) were selected and grown in duplicated 24 deep-well RB blocks (Invitrogen, CS15124) with 4 ml LB Amp^+^ for plasmid extraction and LB Amp^+^ Rha^+^ for protein expression (overnight at 37 °C and 200 rpm). Total protein extraction was performed using B-PER™ (ThermoFischer Scientific, 78248). Briefly, 3 cycles of freezing and thawing of bacterial cell pellet at −80 °C was followed by incubation with B-PER™. Absorption and emission spectra of extracted proteins were measured using VarioSkanTM Lux multimode microplate reader (ThermoFischer Scientific) ranging from 350 to 500 nm with 1 nm step and from 420 to 500 nm with 1 nm step respectively. For each variant, integrated emission intensity was normalized to the corresponding absorbance at 400 nm to calculate relative brightness for further correlation with intracellular brightness. The Ni–NTA purified Electra1 and Elctra2 proteins in 1xPBS were used to record high-quality fluorescence spectra with Steady State Spectrofluorometer FS5 (Edinburgh Instruments).

For expression in *C. elegans*, the target genes were codon-optimized using *C. elegans* codon adapter application (https://worm.mpi-cbg.de/codons/cgi-bin/optimize.py) with insertion of one intron^53^ and de novo synthesized (Tsingke Biotechnology Co., Ltd, China). The optimized genes were cloned into pSF11 vector (Addgene plasmid #159606) under paneuronal promoter regulation *tag*-168. wmScarlet (Psur-5::sur-5::NLSwmScarlet) was used as a reporter marker for screening positive worms (provided by SunnyBiotech Co. Ltd, China). For expression in zebrafish larvae, zebrafish pan-neuronal expression vector with the insert of the BFP-P2A-mScarlet-SV40pA genes under the control of 3.5 kb genomic fragment of *Xenopus* neuron-specific β-tubulin (*nbt/ Xla.Tubb2b*) gene promoter^54^ further flanked by Tol2 recognition sites was cloned and used as described below.

### Mammalian cell culture

HEK293FT cells (Invitrogen) and HeLa cells (ATCC CCL-2) were grown at 37 °C/5% CO_2_ incubator in Dulbecco’s Modified Eagle Medium (DMEM) supplemented with 10% Fetal Bovine Serum (FBS) and 1% penicillin/streptomycin (PS) on a 24-glass bottom well plate (Cellvis, Cat No: P24-0-N) or 35 mm MatTek dishes (MatTek Life Sciences, P35G-1.5-14-C) after Matrigel (BD Biosciences, 356235) coating at 80–90% confluency. Cells were transiently transfected using Hieff Trans Liposomal Transfection Reagent (Yeasen Biotechnology, 40802ES02) with selected blue variants in pHybrid vector according to the manufacture protocol and incubated for 36 h before imaging. The same growth conditions and transfection protocol was used for pAAV-CAG-BFPs-P2A-EGFP plasmids. For OSER assay, HeLa cells were incubated for 12-16 h post-transfection according to the previously described protocol^30,31^.

Procedures involving experimental animals are reported in accordance with Animal Research: Reporting of In Vivo Experiments (ARRIVE) guidelines. All procedures involving mice at Westlake University were conducted in accordance with the US National Institutes of Health Guide for the Care and Use of Laboratory Animals and approved by the Westlake University Committee on Animal Care. For all experiments involving mice throughout, C57BL/6 J strain (supplied by animal facility of Westlake University) was used regardless of sex. Hippocampal neurons were cultured as previously reported^16^. Briefly, the hippocampi from P0 pups were isolated under a stereomicroscope and washed with ice-cold DMEM/High glucose (Servicebio, G4510). The dissected hippocampal tissues were digested with 0.25% trypsin (GIBCO, 25200056) for 12 min at 37 °C, with gentle shaking every 4 min. The digestion was inhibited by DMEM/High glucose supplemented with 10% FBS preheated at 37 °C. The digested tissue was then gently dissociated using Pasteur pipettes with 10 ul tip. The cell suspension was pelleted and resuspended with fresh medium, then filtered through a 40-μm nylon strainer. The dissociated neurons were plated at a density of 150,000 per well on pre-coated coverslips (ThermoFisher Scientific™, 1254580) with Matrigel (BD Biosciences, 356235) in Advanced MEM containing 10% FBS-ES as seeding medium. After 12 h incubation at 37 °C/5% CO_2_ for cell adhesion, half of the medium was replaced by Neurobasal medium (GIBCO, 21103049) supplemented with 1% GlutaMax (GIBCO, 35050061) and 2% B27 (GIBCO, 17504044). AraC (4 μM, Sigma, C6645) was added when glia cell density was 70–80%. Half of the volume medium was removed and replaced with fresh Neurobasal medium per week thereafter. Transduction was performed with AAV-hSyn-BFPs-P2A-EGFP (10^13^ GC/ml for each AAV) at DIV7. Neurons were incubated at 37 °C/5% CO_2_ until imaging on DIV14.

### Cell culture imaging

Intracellular brightness and photostability measurements were carried out under Nikon Ti2-E widefield microscope equipped with Spectra III Light Engine (LumenCore), the ORCA-Flash 4.0 V3 sCMOS camera (Hamamatsu), and 20x/0.75 objective lens controlled by NIS Elements software using BFP (excitation 390/22 nm, emission 457/50 nm), GFP (excitation 475/28 nm, emission 535/46 nm), RFP (excitation 555/28 nm, 594/40 nm) channel. Intracellular photostability measurements were performed under continuous wide-field illumination at light power of 4.25 mW/mm^2^ and 0.91 mW/mm^2^ in HEK cells and 3.72 mW/mm^2^ in neurons. For high-quality images of HeLa cells expressing BFPs fusions Olympus FV3000-IX83 inverted confocal microscope was used, equipped with solid-state diode lasers (OBIS, Coherent) at 405 nm, bandpass exciter filter 360–370 nm, dichroic beam splitter DM410 and BA420-460, multi-alkali PMT (2CH Spectral detector) and U Plan S Apo 20x/0.75 and 40x/0.6 objective lenses. For OSER assay, Nikon Ti2-E widefield fluorescence microscope was used with the same optical configuration as previously stated. Cells were evaluated and counted by three researchers independently and blindly.

### Caenorhabditis elegans transgenic lines preparation and imaging

The transgenic lines were prepared by SunyBiotech Co. Ltd (China) according to standard protocols. Briefly, wild-type N2 worms were co-injected with three plasmids encoding codon optimized genes wBFP::wmNeonGreen::NLSwrmScarlet with final concentrations 10 ng/μl, 10 ng/μl, and 5 ng/μl, respectively. mScarlet was used as selection marker expressed in most somatic cells. Transgenic lines were selected by red fluorescence and confirmed with sequencing of the target transgenes. All worm lines were maintained and selected under GFP channel regardless of blue fluorescence until F4. Lines were subsequently frozen at −80 °C for 3 months according to the standard protocol^55^ using liquid freezing solution containing 129 ml 0.05 M K_2_HPO_4_, 871 ml 0.05 M KH_2_PO_4_, 5.85 g NaCl) + 30%v/v molecular grade glycerol. Upon recovery, worms were grown and maintained at 20 °C on nematode growth media (NGM) plates and fed with OP50 *E. coli* strain as previously reported^56^.

The L4 hermaphrodite adults were transferred on glass slides precoated with 5% agar pads according to previously reported protocol (www.wormatlas.org/agarpad.htm) with a minor modification: 25 mM levamisole (Sangon Biotech Co., Ltd (China) A506644) in M9 was used instead of sodium azide (NaN_3_) to immobilize worms. For brightness quantification, worms were imaged under identical conditions using Nikon Eclipse Ti2-E inverted widefield microscope in blue and green channels. Structural imaging was performed under Olympus FV3000-IX83 inverted confocal microscope, equipped with the 405 nm, 488 nm and 561 nm solid state diode lasers (OBIS, Coherent), bandpass exciter filters 360–370 nm, 460–495 nm, 535–555 nm, dichroic beam splitters DM410, DM505, DM565 and emission filters BA420-460, BA510-550, BA570-625 for blue, green and red, respectively, multi-alkali PMT (2CH Spectral detector) and U Plan S Apo 20x/0.75, 40x/0.6.

### Zebrafish larvae preparation and imaging

All zebrafish experiments were performed as described previously^16^ in accordance with the animal protocol approved by German legislation following European Union guidelines (EU Directive 2010_63). Briefly, pTol2-*nbt*:BFP-P2A-mScarlet plasmid was co-injected with *tol2* mRNA (1.5 nl of injection mix containing 25 ng/μl of both *tol2* and pTol2-plasmid) into one cell stage embryos of the pigmentation-compromised zebrafish *brass* strain. Injection of this construct into zebrafish embryos allows for Tol2-transposon-mediated early genomic integration of the transgene, resulting in broader expression in neurons throughout the brain and spinal cord of larval zebrafish in a semi-mosaic manner. Injected larvae expressing BFPs and mScarlet in the brain and spinal cord at 2–3 days post fertilization (dpf) were sorted using a Leica stereo-fluorescent microscope. Subsequent fluorescence imaging was performed using laser scanning confocal microscope (TCS SP8, Leica Microsystems, Wetzlar Germany) with 40 × NA1.1 water objective. Overview images of zebrafish showing blue fluorescence were acquired (excited by UV 405 nm laser, detection range at 420–480 nm) as multiple *z* stack images using the Tile scan mode and were assembled into tiled images using LAS X software (Leica Microsystems, Wetzlar Germany). For two-color imaging, each of BFPs (excited by UV 405 nm laser, detection range at 420–480 nm) and mScarlet (excited by DPSS 561 nm laser, detection range at 565–606 nm) expressed in neurons in the hindbrain and spinal cord were imaged simultaneously. Reconstructions and projections from z-stacks of images were generated with the 3D-projection program included in the LAS X software. Acquired images were processed with FIJI to measure the fluorescent intensity ratio of each BFP and mScarlet in each neuron. In vivo photostability of each BFP was assessed using spinal cord neurons continuously exposed to UV 405 nm laser set at 10% of the laser power in the software setting. The region of interest (96.88 µm × 96.88 µm) was drawn encompassing spinal cord neurons labeled with each BFP, and single plan image (optical section: 3.56 µm) was recorded every 0.65 s continuously for 3 min. Acquired images were processed with FIJI to measure the fluorescent intensity in each neuron at every time point.

### Virus injection and craniotomy

All the viruses were purchased from Shanghai Sunbio Medical Biotechnology Co., Ltd (China). For protein expression across the cortex, viruses (~ 10^12^ GC/ml) were injected pan-cortically into P0 pups with a Hamilton microliter syringe. To induce hypothermic anesthesia, the pups were put on ice for 5 min until they stopped responding to gentle squeezing on the limbs. For each hemisphere, 0.5 μl of virus solution supplemented with 10% FastGreen dye (SigmaAldrich) was injected under the skull and above the dura manually. After injections on both hemispheres, the pups were moved on a heating pad maintaining 37 °C for a 5-min recovery. After the pups regained responses to gentle squeezing, they were returned to the home cages. The whole injection process for each pup were controlled within few minutes to prevent from cannibalism.

4 weeks after virus injection, the injected mice were anesthetized and fixed onto a stereotaxic station. A circle-shaped craniotomy of 3 mm diameter was applied above the primary somatosensory cortex. Then the craniotomy was enclosed by attaching a custom-made petri dish with a hole on the bottom, to the surface of the skull. A drop of sterile saline was applied to the exposing brain tissue to prevent from drying out. 1 ml of 2% dexamethasone was administered subcutaneously to prevent brain edema.

### Two-photon imaging

After the craniotomy, animals were fixed under the two-photon microscope (Olympus FLUOVIEW, FVMPE-RS). Cells were visualized using a 25 × NA1.05 water objective (Olympus MPXLPlanN). GFP fluorescence was excited at 920 nm and selected by a 495-540 nm bandpass filter. BFP fluorescence was excited at 800 nm and selected by a 410-460 nm bandpass filter. The static images were taken by stepwise scanning 1 μm per step beginning from the dura.

### Data analysis and software

SnapGene was used for in silico cloning and DNA sequence analysis. NIS-Elements Advance Research Software was used for Region of Interest (ROI) manual selection and Mean Fluorescence Intensity (MFI) values extraction throughout experiments, unless stated otherwise. For each field of view (FOV) channel background was subtracted from all MFI values of corresponding channel. Ratio values were calculated in the form of blue-to-red or blue-to-green fluorescence intensity depending on the construct used. For mean brightness calculation in all experiments (except *C. elegans* where all values were included), ratio values greater than Q3 + 1.5xIQR and negative values (or ratios < Q1-1.5 × IQR, if present) were considered as outliers and excluded from graphs. Denoise algorithm of NIS Elements Analysis was used to produce all fusion images and OSER images. Online deconvolution software of NIS Elements (deconv.laboratory-imaging.com/process) was used to produce neuron images with the following parameters: widefield, 20 × magnification, 0.75 NA, 1.00 immersion refractive index, 0.3 calibration, 456 nm channel emission, 519 nm channel1 emission. The PyMol Molecular Graphics System was used to generate structural images. All brightness and photostability graphs as well as statistical analysis were performed and produced on OriginPro (OriginLab, Northampton, MA) except for normalized in vitro brightness and normalized product of brightness and photostability where Excel (Microsoft) was used.

## Supplementary Information

Supplementary Information 1.

Supplementary Information 2.

## Data Availability

All data (raw and analyzed) generated during this study are available from the corresponding author upon request. Genes encoding the two new fluorescent proteins have been deposited in GenBank (Accession Numbers: OL631606 for Electra1, OL631607 for Electra2).
