# Supplementary material for: Dual-expression system for blue fluorescent protein optimization
Source: Sci Rep. 2022 Jun 17;12:10190. doi: 10.1038/s41598-022-13214-0 (PMC9206027; doi:10.1038/s41598-022-13214-0)
Supplement: Supplementary file 2 — Supplementary Information 2. [file 41598_2022_13214_MOESM2_ESM.pdf]

Dual-Expression System for Blue Fluorescent Protein Optimization

Stavrini Papadaki[1,2,3], Xinyue Wang[4], Yangdong Wang[1,2,3], Hanbin Zhang[1,2,3], Su Jia[1,2,3], Shuhong Liu[1,2,3], Minghan Yang[1,2,3,5], Dongdong Zhang[1,2,3], Jie-Min Jia[1,2,3,6], Reinhard W. Köster[4], Kazuhiko Namikawa[4], Kiryl D. Piatkevich[1,2,3]*

[1]School of Life Sciences, Westlake University, Hangzhou, Zhejiang Province, China

[2]Westlake Laboratory of Life Sciences and Biomedicine, Hangzhou, Zhejiang Province, China

[3]Institute of Basic Medical Sciences, Westlake Institute for Advanced Study, Hangzhou, Zhejiang Province, China

[4]Division of Cellular and Molecular Neurobiology, Zoological Institute, Technische Universität Braunschweig, Germany

[5]College of Physics, Jilin University, Changchun, Jilin Province, 130012, China

[6]Key Laboratory of Growth Regulation and Translational Research of Zhejiang Province, School of Life Sciences, Westlake University, Hangzhou, China

Stavrini Papadaki: stapapadaki@westlake.edu.cn

XinyueWang: xinyue.wang@tu-braunschweig.de

Yangdong Wang: wangyangdong@westlake.edu.cn

Hanbin Zhang: zhanghanbin@westlake.edu.cn

Su Jia: iy20310@bristol.ac.uk

Shuhong Liu: shuhong.liu@emory.edu

Minghan Yang: avdymh@gmail.com

Dongdong Zhang: zhangdongdong@westlake.edu.cn

Jie-Min Jia: jiajiemin@westlake.edu.cn

Reinhard W. Köster: r.koester@tu-braunschweig.de

Kazuhiko Namikawa: kaznamik@tu-braunschweig.de; k.namikawa@tu-braunschweig.de

*Kiryl D. Piatkevich: kiryl.piatkevich@westlake.edu.cn

Supplementary Table S1. Correlation of intracellular brightness in HEK293 cells and *in vitro* brightness in E. coli.

Pearson's correlation

	r	R-squared	p-value
Fluorescence in HEK/E.coli	-0.25302	0.0640191204	0.03884

Supplementary Table S2. Correlation of intracellular brightness in HEK293 cells and fluorescence half-time

Pearson's correlation

	r	R-squared	p-value
Fluorescence in HEK/E.coli	0.40639	0.1651528321	6.43948E-4

Supplementary Table S3. Statistics on HEK293 cell culture expressing 7 best performing BFP variants via pHybrid vector.

Descriptive Statistics on blue-to-red intracellular fluorescence ratio

	N total	Mean	Standard Deviation	SE of mean	Variance	Minimum	Q1	Median	Q3	Maximum
**Electra1**	77	9.39774	10.5518	1.20249	111.34039	0.1717	2.11447	4.94095	13.6657	52.32815
**Electra2**	79	8.94347	12.00747	1.35095	144.17934	0.50567	3.05271	5.37942	9.21023	69.47778
**Mutant#1**	52	5.83535	11.00742	1.52645	121.16319	0.18928	1.4586	2.84521	6.39924	75.67304
**Mutant#2**	93	5.98311	4.93338	0.51157	24.33821	0.46764	2.99807	5.05748	7.36312	37.09141
**Mutant#3**	87	6.58148	9.75931	1.04631	95.24415	0.28813	2.36066	4.39454	7.02236	83.36449
**Mutant#4**	75	3.99146	3.88365	0.44845	15.08277	0.40439	1.42278	2.49291	5.49586	18.15178
**Mutant#5**	55	3.38697	3.06371	0.41311	9.38631	0.18457	1.05134	2.25797	5.41468	11.20998

Shapiro-Wilk test of normality on blue-to-red intracellular fluorescence ratio.

	DF	Statistic	p-value	Decision at level (5%)
**Electra1**	73	0.83624	1.60326E-7	Reject normality
**Electra2**	71	0.93268	9.0704E-4	Reject normality
**Mutant1**	48	0.85581	3.11e-5	Reject normality
**Mutant2**	89	0.94808	0.00137	Reject normality

**Mutant3**	80	0.93335	4.31785E-4	Reject normality
**Mutant4**	72	0.83567	1.79077E-7	Reject normality
**Mutant5**	55	0.84605	5.24e-6	Reject normality

Kruskal-Wallis nonparametric analysis of variance on blue-to-red intracellular fluorescence ratio.

	N total	Mean Rank	Sum Rank
**Electra1**	73	280.20548	20455
**Electra2**	71	286.91549	20371
**Mutant1**	48	195.1875	9369
**Mutant2**	89	280.55056	24969
**Mutant3**	80	253.575	20286
**Mutant4**	72	192.19444	13838
**Mutant5**	55	182.32727	10028

Test statistics

Chi-square	DF	p-value
43.7173	6	8.41026E-8

Kolmogorov-Smirnoff test results on pairwise comparison of blue-to-red intracellular fluorescence ratio.

Protein1	Protein2	D	Z	p-value
**ELectra1**	**Mutant #2**	0.22903	1.4504	0.02405
**ELectra2**		0.08799	0.55296	0.88469

Supplementary Table S4. Statistics on HEK293 cells expressing BFPs via P2A self-cleaving peptide.

Descriptive statistics.

Protein	N total	Mean	Standard Deviation	SE of mean	Variance	Minimum	Q1	Median	Q3	Maximum
**mBlueberry2**	853	0.10553	0.01829	6.26288E-4	3.34578E-4	0.05803	0.09474	0.10649	0.11581	0.21499
**EBFP2**	575	0.33929	0.04222	0.00176	0.00178	0.20746	0.31111	0.33951	0.36863	0.48774
**mTagBFP2**	1712	0.48954	0.03016	7.28995E-4	9.09814E-4	0.38097	0.4705	0.48973	0.50786	0.63303
**Electra1**	1403	0.47031	0.08327	0.00222	0.00693	0.37409	0.42502	0.44385	0.47242	0.99216
**Electra2**	714	0.48598	0.05525	0.00207	0.00305	0.39672	0.45577	0.47779	0.49984	0.86273

Shapiro-Wilk test of normality.

	DF	Statistic	p-value	Decision at level (5%)
**mBlueberry2**	835	0.99114	6.5198E-5	Reject normality

**EBFP2**	573	0.99759	0.58299	Can't reject normality
**mTagBFP2**	1698	0.99688	0.00167	Reject normality
**Electra1**	1229	0.91507	0	Reject normality
**Electra2**	664	0.99746	0.40638	Can't reject normality

Kruskal-Wallis nonparametric analysis of variance.

	N total	Mean Rank	Sum Rank
**mBlueberry2**	835	418	349030
**EBFP2**	573	1148.16754	657900
**mTagBFP2**	1698	3736.13133	6343951
**Electra1**	1229	2402.09113	2952170
**Electra2**	664	3304.89307	2194449

Test statistics.

Chi-square	DF	p-value
3698.25584	4	0

Kolmogorov-Smirnoff test results on pairwise comparison of blue-to-green intracellular brightness ratio in HEK293 cells.

Protein 1	Protein 2	D	Z	p-value
**mBlueberry2**	**Electra1**	1	22.29792	0
**EBFP2**		0.88252	17.44611	3.15498E-265
**mTagBFP2**		0.61223	16.34742	6.85708E-233
**mBlueberry2**	**Electra2**	1	19.23209	0
**EBFP2**		0.95582	16.76305	4.59656E-245
**mTagBFP2**		0.20689	4.52021	2.87311E-18
**Electra1**		0.45163	9.37699	4.95764E-77

Linear fit of low-power time dependent fluorescence in HEK293 cells.

Protein	Intercept		Slope		Statistics
	Value	S.E.	Value	S.E.	Adj. R-square
**EBFP2**	97.97858	0.13547	-8.33e-4	2.59991e-4	0.09338
**mTagBFP2**	98.53974	0.23901	-487.4e-4	4.58708e-4	0.99209
**Electra1**	110.07169	0.13845	-282e-4	2.35891e-4	0.99513
**Eelctra2**	103.31536	0.20057	-341.3e-4	3.84935e-4	0.98868

Supplementary Table S5. OSER assay quantification and ratiometric measurements.

Protein	RUN	# Cells with normal ER	# Cells with whorls	# Cells analyzed	% Cells with normal ER
**mTagBFP2**	SP-1	90	21	111	81.08108108
	SP-2	80	13	93	86.02150538
	SP-3	46	25	71	64.78873239
	HBZ-1	17	7	24	70.83333333
	HBZ-2	30	20	50	60
	HBZ-3	28	21	49	57.14285714
	XTS-1	70	26	96	72.91666667
	XTS-2	130	68	198	65.65656566
	XTS-3	119	73	192	61.97916667
**TOTAL**		**610**	**274**	**884**	
**MEAN**					**68.93554537**
**SE**					**3.332631652**

**Electra1**	SP-1	38	13	51	74.50980392
	SP-2	47	7	54	87.03703704
	SP-3	29	17	46	63.04347826
	SP-4	30	19	49	61.2244898
	HBZ-1	23	24	47	48.93617021
	HBZ-2	42	22	64	65.625
	HBZ-3	25	43	68	36.76470588
	HBZ-4	22	26	48	45.83333333
	XTS-1	36	15	51	70.58823529
	XTS-2	56	14	70	80
	XTS-3	59	20	79	74.6835443
	XTS-4	66	18	84	78.57142857
**TOTAL**		**473**	**238**	**711**	
**MEAN**					**65.56810222**
**SE**					**4.560689973**
**Electar2**	SP-1	52	29	81	64.19753086
	SP-2	12	9	21	57.14285714
	SP-3	23	10	33	69.6969697
	SP-4	26	15	41	63.41463415
	HBZ-1	27	29	56	48.21428571
	HBZ-2	18	14	32	56.25
	HBZ-3	6	4	10	60
	HBZ-4	8	4	12	66.66666667
	XTS-1	120	81	201	59.70149254
	XTS-2	85	42	127	66.92913386
	XTS-3	39	24	63	61.9047619
	XTS-4	35	23	58	60.34482759
**TOTAL**		**451**	**284**	**735**	
**MEAN**					**61.20526334**
**SE**					**1.830979185**

RUN represents initials of the experimenters who performed the image analysis.

Ratiometric measurements of whorl structure MFI:NE MFI

	**N total**	**Mean**	**SD**	**SEM**	**Variance**
**EBFP2**	35	2.67025	0.53068	0.0897	0.28163
**mTagBFP2**	33	2.90638	0.7027	0.12232	0.49379
**Electra1**	35	2.76681	0.60467	0.10221	0.36562
**Electra2**	35	2.76112	0.56599	0.09567	0.32035

Two sample t-test

	**T Statistic**	**DF**	**Prob>|t|**
**EBFP2/mTagBFP2**	-1.56945	66	0.12133
**EBFP2/Electra1**	-0.71012	68	0.48006
**EBFP2/Electra2**	-0.6929	68	0.49073

Supplementary Table S6. Statistics on cultured neurons expressing BFPs via rAAV.

Descriptive statistics.

**Protein**	**N total**	**Mean**	**Standard Deviation**	**SE of mean**	**Variance**	**Minimum**	**Q1**	**Median**	**Q3**	**Maximum**
**EBFP2**	36	0.502	0.1371	0.02285	0.0188	0.22179	0.41942	0.50557	0.5925	0.92567
**mTagBFP2**	39	0.7767	0.38474	0.06161	0.14803	0.50525	0.57192	0.65483	0.85467	2.38532
**Electra1**	40	0.56988	0.1576	0.02492	0.02484	0.36438	0.45671	0.55344	0.63678	1.16147

**Electra2**	41	0.63424	0.17101	0.02671	0.02924	0.43029	0.53804	0.58375	0.65041	1.33346

Shapiro-Wilk test of normality.

	DF	Statistic	p-value	Decision at level (5%)
**EBFP2**	35	0.9912	0.99217	Can’t reject normality
**mTagBFP2**	37	0.91925	0.01056	Reject normality
**Electra1**	38	0.96188	0.21844	Can't reject normality
**Electra2**	40	0.73855	4.4416E-7	Reject normality

Kruskal-Wallis nonparametric analysis of variance.

	N total	Mean Rank	Sum Rank
**EBFP2**	35	46.94286	1643
**mTagBFP2**	37	104.97297	3884
**Electra1**	38	63.31579	2406
**Electra2**	40	84.8	3392

Test statistics

Chi-square	DF	p-value
36.97167	3	4.66532E-8

Kolmogorov-Smirnoff test results on pairwise comparison of blue-to-green intracellular brightness ratio on hippocampal neurons.

Protein 1	Protein 2	D	Z	p-value
**EBFP2**	**Electra1**	0.26692	1.13931	0.11917
**mTagBFP2**		0.42105	1.82305	0.00122
**EBFP2**	**Electra2**	0.43929	1.89793	9.05359E-4
**mTagBFP2**		0.30946	1.35672	0.03782
**Electra1**		0.29605	1.3069	0.0501

Supplementary Table S7. Statistics on *C. elegans* expressing BFPs.

Descriptive statistics.

Protein	N total	Mean	Standard Deviation	SE of mean	Variance	Minimum	Q1	Median	Q3	Maximum
**mTagBFP2**	9	0.10464	0.01377	0.00459	1.89725e-4	0.0921	0.09741	0.09946	0.10293	0.13594
**Electra1**	9	0.24848	0.02554	0.00851	6.52345e-4	0.21359	0.23937	0.23937	0.27451	0.28169
**Electra2**	9	0.22883	0.02763	0.00921	7.63442e-4	0.18236	0.22153	0.2343	0.24453	0.26084

Shapiro-Wilk test of normality.

Protein	DF	Statistic	p-value	Decision at level (5%)
**mTagBFP2**	9	0.78111	0.0124	Reject normality
**Electra1**	9	0.90951	0.31248	Can't reject normality
**Electra2**	9	0.87858	0.15169	Can't reject normality

Kruskal-Wallis nonparametric analysis of variance.

	N total	Mean Rank	Sum Rank
**mTagBFP2**	9	5	45
**Electra1**	9	19.88889	179
**Electra2**	9	17.11111	154

Test statistics

Chi-square	DF	p-value
33.84078	2	1.29201e-4

Kolmogorov-Smirnoff test results on pairwise comparison of blue-to-red intracellular brightness ratio on *C elegans*.

Protein 1	Protein 2	D	Z	p-value
**mTagBFP2**	**Electra1**	1	2.12132	4.11353e-5
**mTagBFP2**	**Electra2**	1	2.12132	4.11353e-5
**Electra1**	**Electar2**	0.33333	0.70711	0.73011

Supplementary Table S8. Statistics on zebrafish expressing BFPs and mScarlet (hindbrain and spinal cord).

Descriptive statistics (hindbrain).

Protein	N total	Mean	Standard Deviation	SE of mean	Variance	Minimum	Q1	Median	Q3	Maximum
**mTagBFP2**	120	66.8782	32.50214	2.96703	1056.38893	18.02034	44.06332	59.70697	82.53345	207.41243
**Electra1**	120	53.36642	13.44695	1.22753	180.82055	25.61981	42.38444	53.00699	61.33765	111.44857
**Electra2**	120	75.32743	16.30646	1.48857	265.90077	49.52322	62.42854	72.85091	85.40001	127.55977

Shapiro-Wilk test of normality (hindbrain).

	DF	Statistic	p-value	Decision at level (5%)
**mTagBFP2**	116	0.96464	0.00374	Reject normality
**Electra1**	117	0.98436	0.19288	Can't reject normality
**Electra2**	116	0.96576	0.00463	Reject normality

Kruskal-Wallis nonparametric analysis of variance (hindbrain).

	N total	Mean Rank	Sum Rank
**mTagBFP2**	116	169.72414	19688
**Electra1**	117	116.71795	13656
**Electra2**	116	239.06034	27731

Test statistics

Chi-square	DF	p-value
86.12558	2	1.9864E-19

Kolmogorov-Smirnoff test results on pairwise comparison of blue-to-red intracellular brightness ratio on Zebrafish (hindbrain).

Protein 1	Protein 2	D	Z	p-value
**mTagBFP2**	**Electra1**	0.34505	2.63345	1.15881E-6
**mTagBFP2**	**Electra2**	0.37069	2.82309	1.40923E-7
**Electra1**	**Electra2**	0.59645	4.55215	8.60274E-19

Descriptive statistics (spinal cord).

Protein	N total	Mean	Standard Deviation	SE of mean	Variance	Minimum	Q1	Median	Q3	Maximum
**mTagBFP2**	120	63.84211	27.63005	2.52227	763.4195	28.36681	43.08823	57.21074	77.4192	199.30522
**Electra1**	120	56.50349	9.80906	0.89544	96.2176	36.36839	50.09479	55.43769	61.75298	83.89285
**Electra2**	120	66.6344	12.88311	1.17606	165.97453	38.95269	59.24216	64.32079	70.51041	115.28543

Shapiro-Wilk test of normality (spinal cord)

Protein	DF	Statistic	p-value	Decision at level (5%)
**mTagBFP2**	113	0.95543	8.49921E-4	Reject normality
**Electra1**	118	0.96865	0.00734	Reject normality
**Electra2**	117	0.95244	3.96624E-4	Reject normality

Kruskal-Wallis nonparametric analysis of variance (spinal cord).

	N total	Mean Rank	Sum Rank
**mTagBFP2**	113	161.76106	18279
**Electra1**	118	142.64407	16832
**Electra2**	117	218.93162	25615

Test statistic

Chi-spuare	DF	p-value
36.46497	2	1.20707E-8

Kolmogorov-Smirnoff test results on pairwise comparison of blue-to-red intracellular brightness ratio on Zebrafish (spinal cord).

Protein 1	Protein 2	D	Z	p-value
**mTagBFP2**	**Electra1**	0.26376	2.00395	4.47582E-4
**mTagBFP2**	**Electra2**	0.39914	3.02615	1.26466E-8
**Electra1**	**Electra2**	0.43879	3.36327	1.60383E-10

Supplementary Table S9. Statistics on cortical neurons *in vivo* of blue-to-green fluorescence ratio-two photon microscopy.

Descriptive statistics (L1 and L2/3).

Protein	N total	Mean	Standard Deviation	SE of mean	Variance	Minimum	Q1	Median	Q3	Maximum
**mTagBFP2**	39	3.03754	1.02713	0.16447	1.055	2.02991	2.36311	2.66803	3.53728	7.27518
**Electra1**	44	3.59298	0.80234	0.12096	0.64375	2.55865	3.03282	3.37053	3.98017	6.04244
**Electra2**	41	2.59075	0.39813	0.06218	0.15851	1.92311	2.31204	2.48427	2.86459	3.44724

Shapiro-Wilk test of normality (L1 and L2/3).

	DF	Statistic	p-value	Decision at level (5%)
**mTagBFP2**	38	0.8931	0.00164	Reject normality
**Electra1**	43	0.90954	0.00247	Reject normality
**Electra2**	38	0.94807	0.0769	Can’t reject normality

Kruskal-Wallis nonparametric analysis of variance (L1 and L2/3).

	N total	Mean Rank	Sum Rank
**mTagBFP2**	38	54.28947	2063
**Electra1**	43	85.46512	3675
**Electra2**	38	36.89474	1402

Test statistic

Chi-spuare	DF	p-value
41.52093	2	9.63484E-10

Kolmogorov-Smirnoff test results on pairwise comparison of blue-to-green fluorescence ratio in live cortical neurons-two photon microscopy (L1 and L2/3).

Protein1	Protein2	D	Z	p-value
**mTagBFP2**	**Electra1**	0.53244	2.39139	8.3619E-6
	**Electra2**	0.26316	1.14708	0.14451
**Electra1**	**Electra2**	0.69951	3.14179	5.66308E-10

Descriptive statistics (L1 and L2/3 independently).

Protein	Layer	N total	Mean	Standard Deviation	SE of mean	Variance	Minimum	Q1	Median	Q3	Maximum
**mTagBFP2**	1	13	4.0945	1.13121	0.31374	1.27964	2.57139	3.53728	3.90341	4.25587	7.27518
	2/3	26	2.50907	0.3435	0.06737	0.11799	2.02991	2.21067	2.49736	2.69554	3.41571
**Electra1**	1	17	4.37951	0.72951	0.17693	0.53218	3.53974	3.85099	4.14103	4.5943	6.04244
	2/3	27	3.09776	0.27928	0.05375	0.078	2.55865	2.87998	3.12135	3.35538	3.54593
**Electra2**	1	21	2.7543	0.36544	0.07975	0.13355	2.12771	2.48427	2.71593	2.9904	3.44724
	2/3	20	2.41902	0.36403	0.0814	0.13251	1.92311	2.29088	2.35933	2.51978	3.23772

Shapiro-Wilk test of normality (L1 and L2/3 independently).

	Layer	DF	Statistic	p-value	Decision at level (5%)
**mTagBFP2**	1	12	0.96836	0.89291	Can't reject normality
	2/3	26	0.95297	0.27195	Can't reject normality
**Electra1**	1	16	0.89992	0.08014	Can't reject normality
	2/3	27	0.96114	0.39216	Can't reject normality
**Electra2**	1	21	0.96102	0.53684	Can't reject normality
	2/3	17	0.88421	0.03722	Reject normality

Kruskal-Wallis nonparametric analysis of variance (L1).

	N total	Mean Rank	Sum Rank
**mTagBFP2**	12	30.83333	370
**Electra1**	16	37.875	606
**Electra2**	21	11.85714	249

Test statistic

Chi-spuare	DF	p-value
32.75761	2	7.70505E-8

Kolmogorov-Smirnoff test results on pairwise comparison of blue-to-green fluorescence ratio in live cortical neurons-two photon microscopy (L1).

Protein1	Protein2	D	Z	p-value

**mTagBFP2**	**Electra1**	0.375	0.98198	0.23709
	**Electra2**	0.83333	2.30283	4.94903E-6
**Electra1**	**Electra2**	1	3.01348	1.5533E-10

Kruskal-Wallis nonparametric analysis of variance (L2/3).

	N total	Mean Rank	Sum Rank
**mTagBFP2**	26	27.88462	725
**Electra1**	27	54.51852	1472
**Electra2**	17	16.94118	288

Test statistic

Chi-spuare	DF	p-value
41.35814	2	1.04519E-9

Kolmogorov-Smirnoff test results on pairwise comparison of blue-to-green fluorescence ratio in live cortical neurons-two photon microscopy (L2/3).

Protein1	Protein2	D	Z	p-value
**mTagBFP2**	**Electra1**	0.73362	2.66994	2.86914E-7
	**Electra2**	0.45928	1.47248	0.01601
**Electra1**	**Electra2**	0.96296	3.11021	5.24509E-11

Supplementary Table 10. Correlation of intracellular brightness in HEK293 cells and molecular brightness for mBlueberry2, EBFP2, mTagBFP2.

Pearson's correlation

	r	R-squared	p-value
Fluorescence in HEK/Molecular Brightness	-0.78501	0.6162407	0.42531

Supplementary Figure S1. Amino acid sequence alignment of the selected BFPs with their parental protein mRuby3 and mTagBFP2.

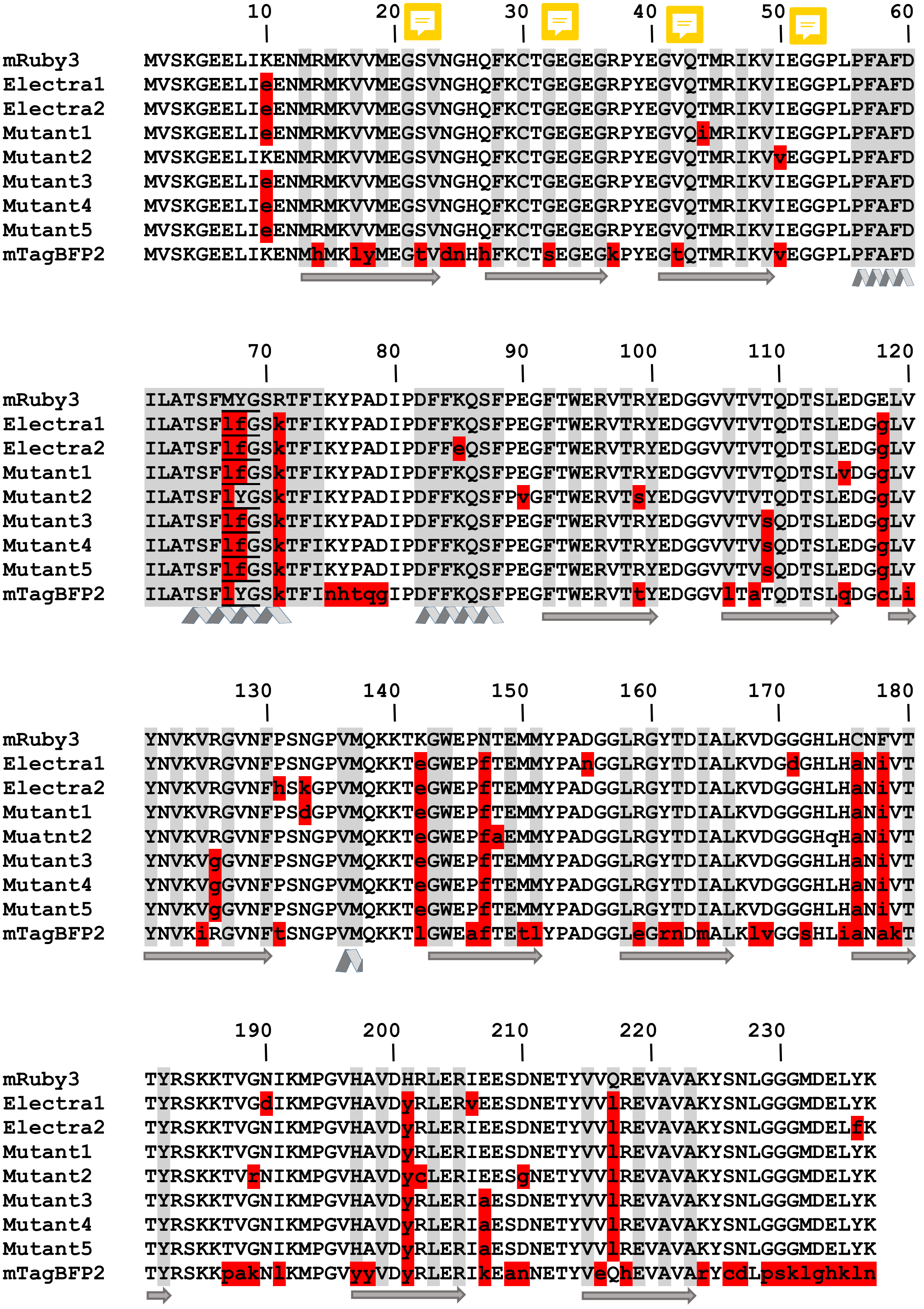

Amino acids forming the chromophore are underlined; single amino acid changes relative to parental mRuby3 are highlighted in red. The β-sheet forming regions and α-helices are denoted with arrows and ribbons, respectively. Amino acids highlighted in grey correspond to internal residues.

Supplementary Figure S2. Electra1 and Electra2 predicted structures.

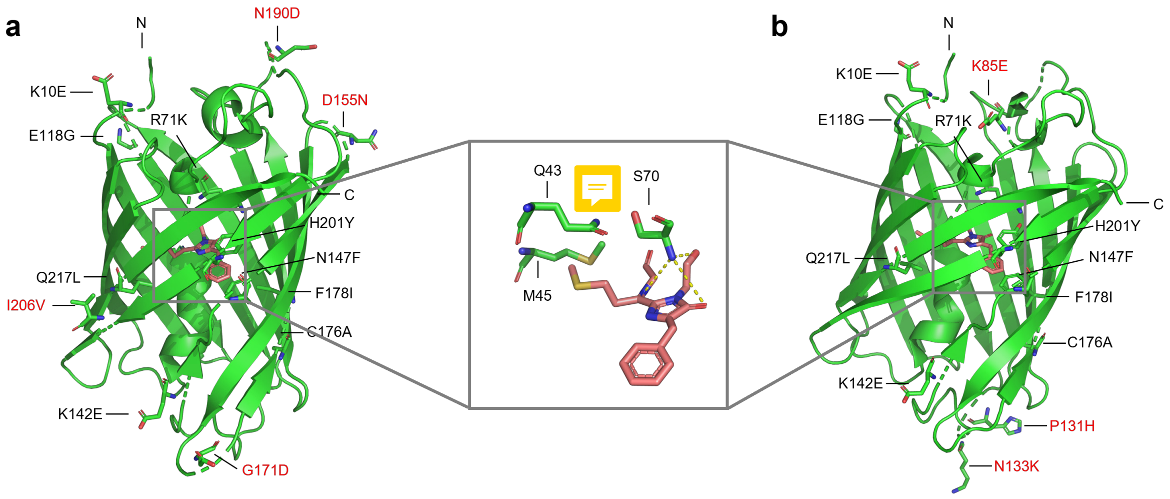

Locations of introduced mutations in (**a**) Electra1 and (**b**) Electra2 with zoom in view of the chromophore with amino acid residues in the proximity to the N-acylimine group (3.5 Å) modeled on the crystal structure of mCRISPRed (D subunit from PDB 6XWY); mutations compared to mRuby3, numbering based on the mRuby3 sequence; chromophore shown in red; common mutations between the two mutants are shown in black font; unique mutations are shown in red font (Electra2 Y236F is not included since mCRISPRed sequence is truncated at amino acid position 226 of mRuby3; chromophore mutations cannot be incorporated as mCRISPRed structure uses NRQ as chromophore). Structures produced using PyMol.

Supplementary Figure S3. Co-expression of the selected BFPs with EGFP in HEK cells.

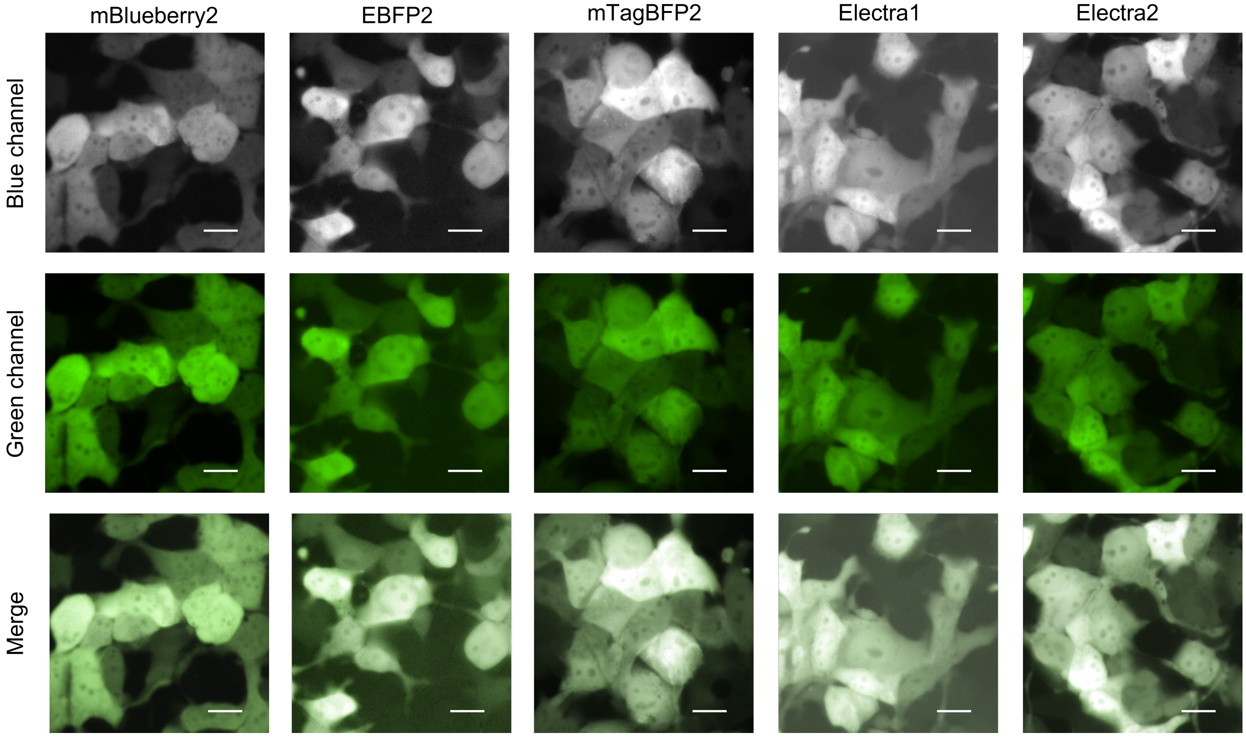

Representative HEK293FT cells co-expressing mBlueberry2, EBFP2, mTagBFP2, Electra1 and Electra2 (white) with EGFP (green) via P2A self-cleaving peptide. Imaging conditions for BFPs: 403 nm LED excitation, emission 456nm, 0.91 mW/mm^2 power. The dynamic range for each image was adjusted independently to facilitate visualization. Scale bars, 20 µm.

Supplementary Figure S4. Photobleacing curves for the selected BFPs.

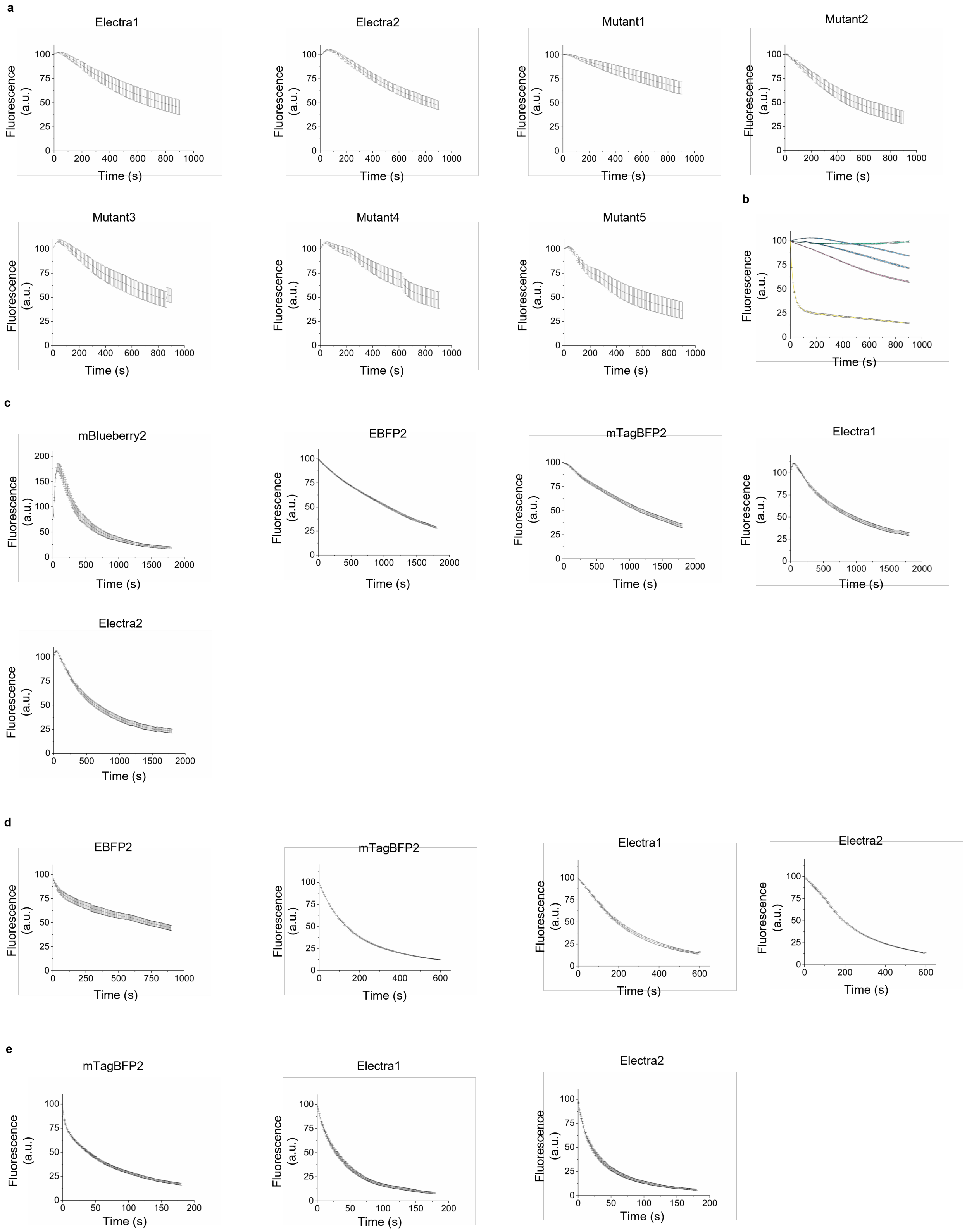

Normalized photobleaching curves for the selected BFPs recorded in live cells under continuous illumination represeted with standard error of mean (SEM). (**a**) Photobleaching curves measured during screening in HEK cells using pHybrid expression vector for Electra1, Electra2, Mutant1, Mutant2, Mutant3, Mutant4, and Mutant 5 (representation of the data with SEM from **Fig. 2c**). (**b**,**c**) Photobleaching curves for mBlueberry2 (yellow), EBFP2 (green), mTagBFP2 (pink), Electra1 (light blue) and Electra2 (dark blue) expressed in HEK cells via P2A self-cleaving peptide under (**b**) low power illumination and (**c**) high power illumination (representation of the data with SEM from **Fig. 3b** and **c**, respectively). (**d**) Photobleaching curves recorded in cultured neurons for EBFP2, mTagBFP2, Electra1, and Electra2 (representation of the data with SEM from **Fig. 5c**). (**e**) Photobleaching curves recorded for mTagBFP2, Electra1 and Electra2 expressed in spinal cord neurons of zebrafish (representation of the data with SE from **Fig. 6f**).

Supplementary Figure S5. Expression of mitochondria localized Electra2 in HeLa cells.

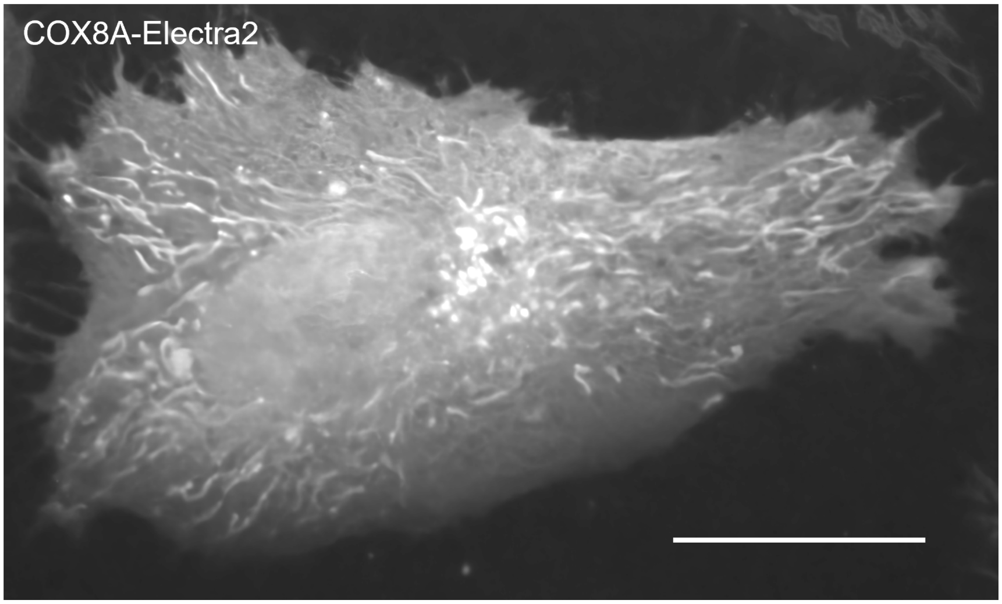

Representative image of HeLa cell expressing the COX8A-Electra2 fusion. Scale bars, 20µm.

Supplementary Figure S6. *In vivo* two-photon microscopy of cortex neurons co-expressing mTagBFP2, Electra1, or Electra2 with EGFP in live mice.

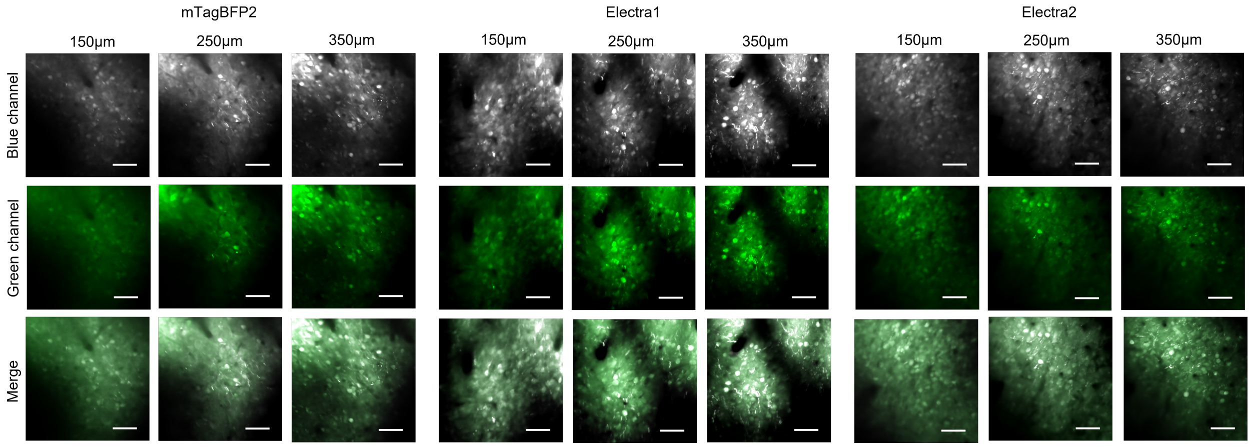

Representative images of mTagBFP2, Electra1, Electra2 from cortex layer1 and layer2/3 (150μm/250μm/350μm depth). Scale bars, 50 μm.

Supplementary Figure S7. Expression of the selected BFPs in mouse brain.

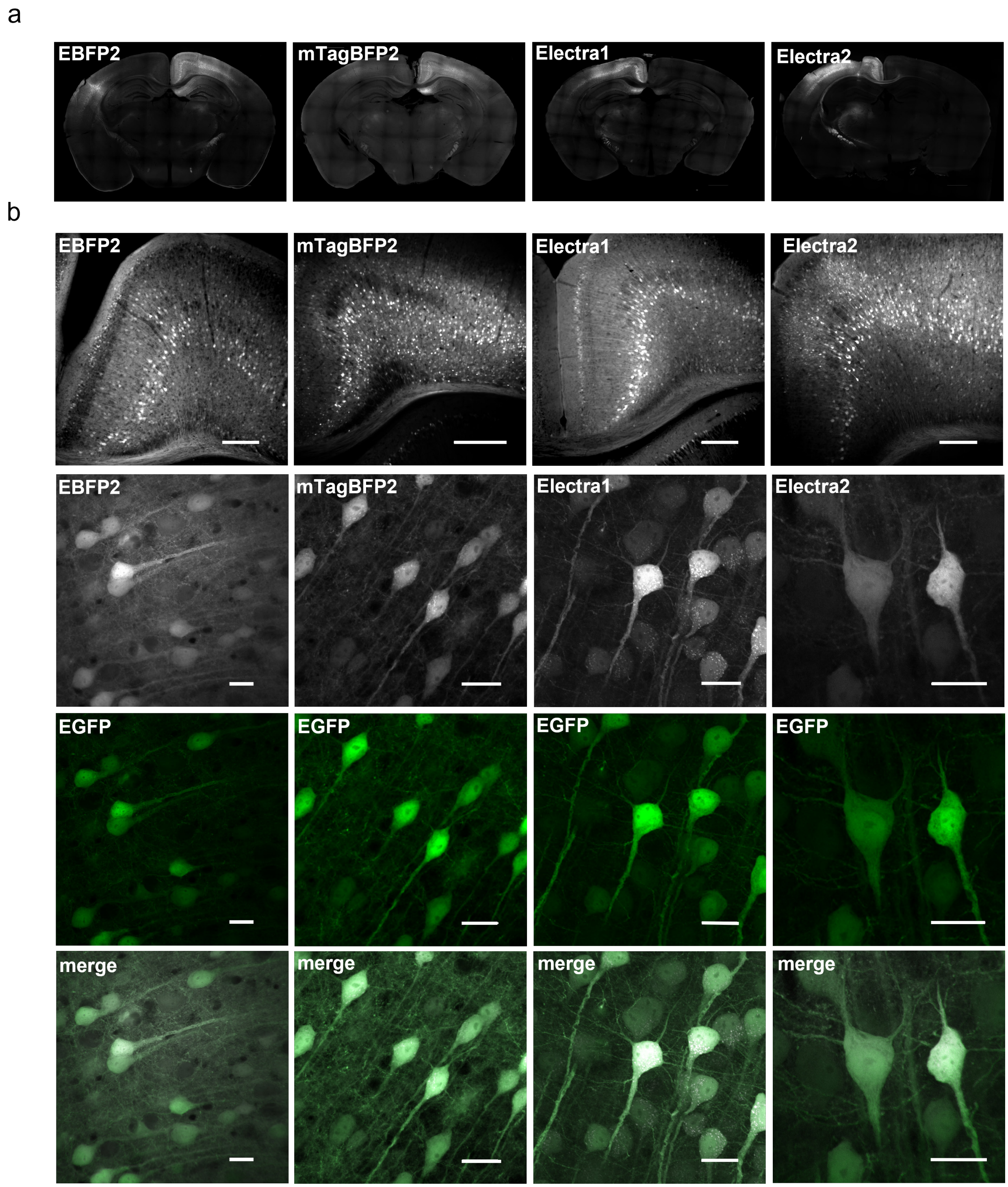

Brightness comparison in fixed mouse brain slices. (**a**) Complete brain slice expressing EBFP2, mTagBFP2, Electra1 and Electra2 at DIV14-21. (**b**) Representative high-resolution images for each protein. BFPs are shown in grey; EGFP in green. Scale bars 20μm.
